# Management of Suspected Cases of Feline Immunodeficiency Virus Infection in Eurasian Lynx (*Lynx lynx*) During an International Translocation Program

**DOI:** 10.3389/fvets.2021.730874

**Published:** 2021-10-25

**Authors:** Marie-Pierre Ryser-Degiorgis, Iris Marti, Simone R. R. Pisano, Mirjam Pewsner, Martin Wehrle, Christine Breitenmoser-Würsten, Francesco C. Origgi, Anna Kübber-Heiss, Felix Knauer, Annika Posautz, Matthias Eberspächer-Schweda, Jon B. Huder, Jürg Böni, Jakub Kubacki, Claudia Bachofen, Barbara Riond, Regina Hofmann-Lehmann, Marina L. Meli

**Affiliations:** ^1^Institute for Fish and Wildlife Health, Vetsuisse Faculty, University of Bern, Bern, Switzerland; ^2^Natur- und Tierpark Goldau, Goldau, Switzerland; ^3^Foundation KORA (Carnivore Ecology and Wildlife Management), Muri bei Bern, Switzerland; ^4^Research Institute of Wildlife Ecology, University of Vienna, Vienna, Austria; ^5^Dentistry and Oral Surgery Service, Department/Hospital for Companion Animals and Horses, University of Veterinary Medicine Vienna, Vienna, Austria; ^6^Swiss National Center for Retroviruses, Institute of Medical Virology, University of Zurich, Zurich, Switzerland; ^7^Institute of Virology, Vetsuisse Faculty, University of Zurich, Zurich, Switzerland; ^8^Clinical Laboratory, Department of Clinical Diagnostics and Services, and Center for Clinical Studies, Vetsuisse Faculty, University of Zurich, Zurich, Switzerland

**Keywords:** animal welfare, *Lynx lynx*, FIV, conservation, decision scheme, infectious disease, wildlife translocation, Switzerland

## Abstract

The Eurasian lynx (*Lynx lynx*) population in Switzerland serves as a source for reintroductions in neighboring countries. In 2016–2017, three lynx from the same geographical area were found seropositive for feline immunodeficiency virus (FIV) in the framework of an international translocation program. This novel finding raised questions about the virus origin and pathogenicity to lynx, the emerging character of the infection, and the interpretation of serological results in other lynx caught for translocation. Archived serum samples from 84 lynx captured in 2001–2016 were retrospectively tested for FIV antibodies by Western blot. All archived samples were FIV-negative. The three seropositive lynx were monitored in quarantine enclosures prior to euthanasia and necropsy. They showed disease signs, pathological findings, and occurrence of co-infections reminding of those described in FIV-infected domestic cats. All attempts to isolate and characterize the virus failed but serological data and spatiotemporal proximity of the cases suggested emergence of a lentivirus with antigenic and pathogenic similarities to FIV in the Swiss lynx population. A decision scheme was developed to minimize potential health risks posed by FIV infection, both in the recipient and source lynx populations, considering conservation goals, animal welfare, and the limited action range resulting from local human conflicts. Development and implementation of a cautious decision scheme was particularly challenging because FIV pathogenic potential in lynx was unclear, negative FIV serological results obtained within the first weeks after infection are unpredictable, and neither euthanasia nor repatriation of multiple lynx was acceptable options. The proposed scheme distinguished between three scenarios: release at the capture site, translocation, or euthanasia. Until April 2021, none of the 40 lynx newly captured in Switzerland tested FIV-seropositive. Altogether, seropositivity to FIV was documented in none of 124 lynx tested at their first capture, but three of them seroconverted in 2016–2017. Diagnosis of FIV infection in the three seropositive lynx remains uncertain, but clinical observations and pathological findings confirmed that euthanasia was appropriate. Our experiences underline the necessity to include FIV in pathogen screenings of free-ranging European wild felids, the importance of lynx health monitoring, and the usefulness of health protocols in wildlife translocation.

## Introduction

Feline immunodeficiency virus (FIV) is a retrovirus of the genus Lentivirus, which is closely related to human immunodeficiency virus causing the acquired immunodeficiency syndrome in humans and characterized by similar disease mechanisms (1–4). FIV infection occurs in domestic cat populations worldwide, and the virus is categorized in different subtypes. In Europe, subtypes A and B are predominant in the North while B occurs more frequently in the South. Subtype A is the most common subtype in Switzerland and Germany, while in southern Europe and Turkey, subtype B is more frequent (5). The worldwide frequency of infection was estimated to vary from 1 to 16% in healthy cats to 44% in sick cats (3), but current data is missing for many regions.

Feline immunodeficiency virus and the closely related puma lentivirus clade A (PLVA) and highly divergent clade B (PVLB) have been detected in numerous wild felids, and species-specific strains have been characterized, but this does not exclude occasional interspecific transmission (6–12). Occurrence of disease signs or pathological changes doubtlessly associated with FIV infection has not been documented in wildlife, however, demonstrating the association of disease with FIV is a challenge that is also encountered with domestic cats (13). Disease signs and lesions commonly associated with FIV include anorexia, lymphadenopathy, blood value alterations, disorders of the digestive and respiratory tract, neurological conditions, and neoplasia (see Supplementary Material 1 for more details).

Serology is the method of choice to detect FIV infection in domestic cats because except for rare cases, all FIV-infected cats develop a strong and persistent antibody response to the virus that remains detectable during the entire infection course. By contrast, virus detection by molecular methods is limited by the genetic plasticity of the virus and very low virus loads during the asymptomatic phase of infection. The latter also hampers virus isolation by cell culture. Western blot (WB) is considered the gold standard for detection of antibodies against FIV (5, 14). Different WB methods have been described (15, 16). In our hands, a WB result is judged positive if two bands with a molecular weight of 15 kDa (capsid protein p15) and 24 kDa (matrix protein p24) are recognizable on the blotting strip (15). Additional information on FIV is provided in Supplementary Material 1.

In Europe, FIV infections in wildlife have not been reported so far. Targeted studies in wildcats (*Felis sylvestris*) (17, 18), Iberian lynx (*Lynx pardinus*) (19, 20), and Eurasian lynx (*Lynx lynx*) (21) have not detected antibodies against FIV by WB. Three antibody-positive wildcats have been reported in France, but they were diagnosed only by enzyme-linked immunosorbent assay (ELISA) (22) and are questionable because they were not confirmed by WB, which is the recommended procedure in case of low prevalence in a population (13). In Switzerland, until 2014 lynx were tested for FIV antibodies by ELISA and no positive result was ever obtained. Based on this apparent absence of FIV in the Swiss lynx population and in European free-ranging wild felids in general, as well its occurrence in other wild felids and its prevalence and pathogenicity in domestic cats worldwide including Swiss cats, it was assumed that the Eurasian lynx may be susceptible to infection and potentially develop disease. Therefore, FIV seropositivity by WB was set as an exclusion criteria for translocation programs (23). As ELISA might not detect antibodies to all FIV subtypes or strains (13), testing with WB was implemented.

In the framework of lynx translocations from Switzerland to Austria and Germany in 2016–2020 (24, 25), three lynx were found positive to FIV by WB during quarantine. A decision scheme was required to minimize health risks both in the recipient and source populations, considering animal welfare (no prolonged stay in quarantine), the political situation (no repatriation to the source population), and conservation goals (preservation and use of the source population for translocations).

The aim of this article is to report the observations made on the three FIV-seropositive cases and to share the rationale behind the implemented disease management measures, and the scheme upon which decision on the fate of these animals was taken. Because getting an overview of the FIV situation in the whole population was a necessary step for decision-making, and continued surveillance essential to assess the development of the situation, we also present WB results of our retrospective and prospective FIV serosurvey.

## Materials and Methods

### Study Areas

**Figure 1:**
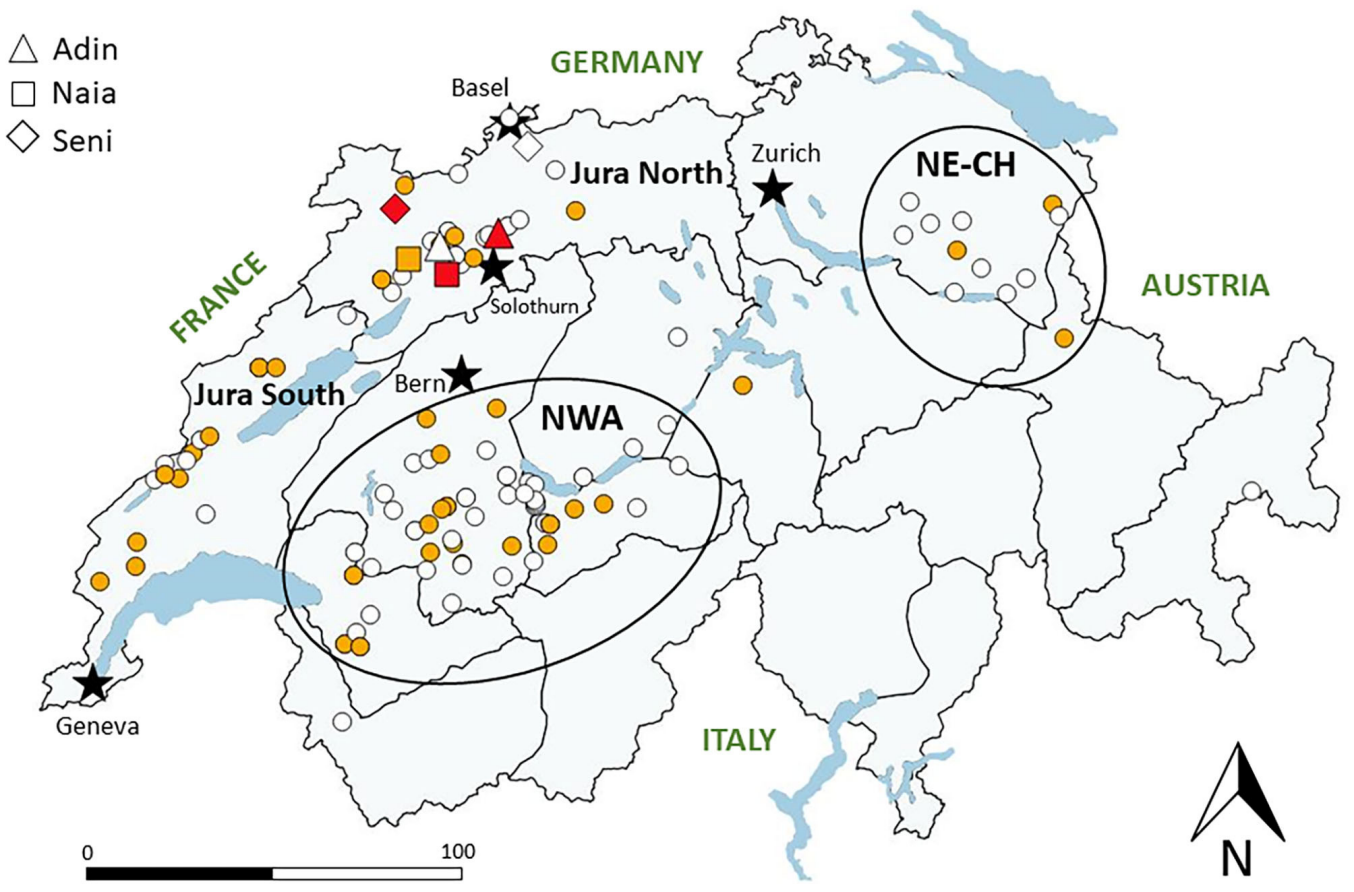
Map of Switzerland with main lakes (blue areas) and five main cities (black stars) depicting the localization of lynx tested for FIV antibodies by Western blot from 2001 to 2021. White: FIV-seronegative (no band); yellow: FIV-negative but p24 band reaction (unspecific cross-reaction, early or late FIV infection phase); red: FIV-seropositive (both p24 and p15 bands). ADIN, NAIA, and SENI are indicated twice (before and after seroconversion), while all other tested lynx (dots) appear only once (first test). Gray lines indicate borders of current lynx management compartments, and black circles indicate the relevant Alpine subpopulation (north-western Alps, NWA) and the more recent small population in north-eastern Switzerland (NE-CH). Jura North and Jura South refer to the lynx management compartment corresponding to the Swiss part of the Jura lynx population.

Samples used in this study originated from the three currently existing Swiss lynx populations, which are further divided in management compartments: Jura South and Jura North, north-western Swiss Alps and other Alpine compartments, and north-eastern Switzerland (Figure 1).

### Animals

This study included a total of 124 wild-born lynx of various ages and both sexes (Table 1), and three adult zoo lynx sampled before being euthanized for population control. All wild-born lynx were captured, examined by a field veterinarian, and sampled under anesthesia from 2001 to 2021 following the same field procedures (23, 26), with the necessary authorizations of the Federal Office of the Environment (capture of a species protected by federal law; permits issued on 17.01.2001, 314.12.2001, 05.09.2002, 26.01.2004, 24.01.2007, 01.03.2011, 17.12.2013, 30.08.2017, 22.07.2020) and of the commission for animal experimentation required in Switzerland to immobilize and sample protected wildlife for research purposes (permits Nr 66/97, 8/00, 89/02 360.431.00, SO57052, BE12/07, 109/10, 111/13+, 3/17, 3/17+, BE61/20). They comprised lynx brought to a quarantine station prior to translocation (*n* = 29), lynx orphans at wildlife rehabilitation centers (*n* = 21), and lynx captured for other purposes such as research and population monitoring (*n* = 74). There were 84 lynx captured from 2001 to 2016, when the first case was diagnosed, and 40 lynx captured for the first time from 2017 to 30 March 2021.

**Table 1:** Wild-born Eurasian lynx (*Lynx lynx*) tested for exposure to feline immunodeficiency virus by Western blot at their first capture in Switzerland, 2001–2021.

**Study period**	**Juvenile (Newborn^**a**^)** ***n*** **= 36 (10)**		**Subadult** ***n*** **= 17**		**Adult** ***n*** **= 71**		**Total *n* = 124**
	**Male**	**Female**	**Male**	**Female**	**Male**	**Female**	
Retrospective testing 2001–2016 (total tested)	9 (4)	18 (4)	6	5	24	22	84
Negative	7 (2)	15 (3)	5	3	16	11	57
p24 band reaction	2 (2)	3 (1)	1	2	8	11	27
Prospective testing 2017–2021 (total tested)	8 (1)	1 (1)	2	4	14	11	40
Negative	6 (1)	1 (1)	1	2	3	5	18
p24 band reaction	2 (0)	0	1	2	11	6	22
Total lynx tested	17 (5)	19 (5)	8	9	38	33	124

*None of them was positive (requiring both a p24 and p15 band reaction), but the table distinguishes animals with a (possibly unspecific) p24 band reaction from those without reaction*.

a *Approximately 4 weeks old (a very young orphan and nine newborn kittens marked at the den)*.

The free-ranging lynx included three FIV-seropositive lynx, all older than 1 year (the two males ADIN and SENI, and the female NAIA). They were trapped with a box trap (ADIN) or foot snares (NAIA and SENI) and anesthetized in the compartment Jura North in 2016 (ADIN) and 2017 (SENI and NAIA). All three had already been captured, examined by a field veterinarian, sampled, and radio-marked for research or conservation purposes before the beginning of the mentioned translocation projects, in 2013 (ADIN) and 2016 (SENI, NAIA). Each of the three lynx underwent at least three clinical examinations and testing (before the project, at capture before being transferred to a quarantine station, and right before euthanasia; Table 2). SENI and NAIA were additionally examined once during quarantine in Switzerland but not ADIN, which was the first FIV-seropositive lynx and was quarantined in Austria due to logistic constraints. Detailed clinical data on ADIN at the time of euthanasia were not available for this study.

**Table 2:** Overview of clinical findings and Western blot results at different time points of the three Eurasian lynx (Lynx lynx) seropositive for feline immunodeficiency virus: 1^st^ field capture (C1), 2^nd^ field capture (C2), during quarantine (Q), and before euthanasia (E). Empty cells: not detected.

**Abnormalities^**a**^**	**ADIN (Male)**			**NAIA (Female)**				**SENI (Male)**			
	**C1**	**C2**	**E^**b**^**	**C1**	**C2**	**Q**	**E**	**C1**	**C2**	**Q**	**E**
Date	14.03.13	19.03.16	24.04.16	13.04.16	09.02.17	07.03.17	21.03.17	20.02.16	17.2.17	07.03.17	21.03.17
Body weight (kg)	24.0	23.0	21.9	16.7	17.2		16.7	14.2	18.2	18.7	17.7
Poor body condition		+		++		+	+		++	+	++
Lymphadenomegaly							+			++	++
Gingiva lesions		++	+++	+	(+)				(+)	+	++
Ocular secretions		(+)				+	(+)				
Purulent rhinitis										+	
Reduced skin turgor		+		+	(+)	+	(+)			+	+
Blood smears, anus					+	++	+		+		+
Feces smears, anus					+				(+)		
Hypotrichosis						+	+	++	+	++	+++
Recent skin wounds		+	n/a	+	+		+	+	++	+	
Acquired dental lesions^c^		+	+++			++				+	
Claws split	+	+	n/a			+	(+)		+	+++	++
Pregnancy	n/a	n/a	n/a	+				n/a	n/a	n/a	n/a
Ectoparasites^d^	++	(+)	n/a	++	+			++	+		
FIV Western blot	negative	positive	positive	p24	positive	positive	p24	Negative	positive	p24	p24

*n/a, not applicable*.

a *Graded as minimal (+), mild +, moderate ++, or severe +++*.

b *Findings at necropsy for comparison (no clinical examination at this date)*.

c *New since previous examination*.

d *Ticks and/or ear mites (Otodectes cynotis)*.

### Clinical Observations

All lynx included in this study were submitted to a standard clinical examination and sample collection by a field veterinarian during anesthesia (23). Blood was drawn from the cephalic vein and collected into sterile EDTA tubes (for hematology and molecular diagnostics) and uncoated tubes (to obtain serum for blood chemistry and serology) using a vacutainer. Blood smears and serum aliquots were prepared within a few hours after capture. Additionally, swabs were taken from several body sites for molecular diagnostics for a series of antigen tests, including feline herpesvirus (FHV) (conjunctiva), feline calicivirus (FCV), and canine distemper virus (CDV) (oropharynx) and feline parvovirus (FPV) and feline coronavirus (FCoV) (rectum). All samples were immediately sent to the laboratory for analysis.

Wild-born lynx in provisory captivity (orphans in rehabilitation center or older lynx in quarantine station) were monitored by regular visual checks including evaluation of food intake and defecation. A few orphans requiring health care were anesthetized and examined more closely multiple times. Lynx to be translocated were submitted to a post-quarantine clinical examination right before transport. NAIA and SENI were additionally monitored by video cameras around the clock and re-examined and re-sampled under anesthesia 4 and 3 weeks after their arrival, respectively, and again at the time of euthanasia. ADIN was also blood sampled before he was euthanized.

### Captive Conditions

Except for a few orphans, lynx were kept in individual enclosures. However, due to logistic constraints, SENI was transferred to NAIA's enclosure once he was also diagnosed FIV-seropositive (see Results).

For the most, lynx enclosures were distant from regular human disturbance. The ground was covered by wood chips or pieces as used for zoo animals and the enclosure was structured with platforms and vegetation providing hides and climbing opportunities. Lynx were fed carcasses (apparently healthy road kills) of wild ungulates belonging to their natural prey spectrum, at a rhythm of about a carcass per week, which corresponds to their normal food intake (27). ADIN was quarantined in another institution in an enclosure not previously adapted to fulfill housing requirements of wild-born lynx. This enclosure consisted of two adjacent horse boxes with concrete ground and concrete hind walls, linked to each other by an opening that could be closed by a sliding door actuated by means of a wire rope, in which ADIN already bite upon arrival. The upper part of the box walls was made of thick metal bars, later covered by a tarp. There were neither hides, nor climbing opportunities except for a small platform surrounded by wood chips. Offered food, which consisted of various animals fed in zoos including slaughtered livestock and poultry chicks, was also inadequate for a wild-born lynx used to prey on wild ungulate. Human disturbance was limited to zoo personnel in charge of the animals during working hours.

### Blood Profiles and Chemistry, Serology, and Molecular Diagnostics for Pathogens

Blood samples for hematology, blood chemistry, serology and molecular diagnostics, as well as all swabs were analyzed for selected pathogens (see above, section “Clinical observations”) as previously described (23). The protocol developed for translocations also required that right after capture, lynx blood had to be tested with a point-of-care (POC) test for FIV antibody and feline leukemia virus (FeLV) antigen detection validated for domestic cats (SNAP FIV/FeLV Combo Test, IDEXX, Switzerland). If the POC test was negative, lynx were brought to quarantine for observation and further testing for a range of pathogens including FIV by WB (23).

After direct release on site (lynx not translocated) or during their stay in quarantine (translocation programs), blood samples of the captured lynx were re-rested by FIV ELISA and/or WB as previously described (5). A quantitative real-time PCR assay (qPCR) to detect FIV provirus (gag region of FIV subtype A and B) was also applied on samples of part of the lynx, including 26 WB-negative and the three positive animals as previously described (5, 28).

Samples of the three FIV seropositive animals were further analyzed. A FeLV ELISA to detect p27 antigen and a FeLV p15E ELISA to detect transmembrane antibodies in serum were performed (29, 30). Serum samples of the first and second capture were also tested for FHV antibodies by indirect immunofluorescence assay (IFA) (31). Whole blood samples were molecularly tested for the presence of FeLV, FHV, CDV, hemotropic mycoplasma species, *Anaplasma phagocytophilum*, and *Rickettsia* species. Fecal swab samples were tested for FPV and FCoV. Oropharyngeal swab samples were tested for FCV, FHV, and CDV. Conjunctival and nasal swab samples were tested for *Chlamydia felis, Mycoplasma felis*, and FHV. Molecular testing was performed by qPCR and/or RT-qPCR as previously described (20, 32, 33).

Total nucleic acids (TNA) from blood samples of the three FIV-seropositive lynx were additionally sent to an external laboratory for FIV provirus PCR diagnosis (IDEXX Diavet AG, Bäch, Switzerland) and also tested using a commercially available real-time RT-qPCR kit (FTvet Feline Anemia I, Fast Track Diagnostics, Esch-sur-Alzette, Luxembourg). These samples were tested using additional conventional nested/semi-nested PCR assays that detect all major FIV subtypes (A, B, and C) (4, 34–36). For more details, refer to Supplementary Material 2.

### Pathology

The three FIV-seropositive lynx were euthanized in agreement with all responsible authorities. Euthanasia was carried out under anesthesia with pentobarbital (Esconarkon^®^, Streuli Tiergesundheit AG, Uznach, Switzerland) injected into the cephalic vein. For the two animals kept in the quarantine station in Switzerland (NAIA and SENI), samples for virological investigations (bone marrow by needle punction, whole blood, serum, nasal, oropharyngeal, and fecal swabs) were taken shortly before Pentobarbital injection. After euthanasia, spleen and lymph nodes from different locations were collected into sterile 50 ml falcon tubes containing 20 ml of cell culture medium (DMEM supplemented with 10% FCS, 1% essential amino acids, 1% ciprofloxacin, 1.5% HEPES; Gibco, ThermoFischer Scientific, Basel, Zurich and Ecublens, Switzerland) and kept at room temperature for approximately 6 h before further processing (as described in Supplementary Material 2). Animals were finally submitted to necropsy. Macroscopic examination was performed according to the standards of the Research Institute of Wildlife Ecology at the University of Vienna (ADIN) and the protocols of the Center for Fish and Wildlife Health at the University of Bern (NAIA, SENI) (23). The following tissues were sampled for histopathology: lung, spleen, lymph nodes, liver, kidney, skeletal muscle, stomach, adrenal gland (all); brain, heart, thyroid gland, urinary bladder, small and large intestine (NAIA, SENI); testicle (ADIN, SENI); ovary and uterus (NAIA); skin, eye, and pancreas (SENI). These were fixed in 10% buffered formalin, embedded in paraffin, sectioned, and stained with hematoxylin-eosin following the accredited protocols of the Institute of Veterinary Pathology at the University of Bern. Additionally, dentition and jaw bones of ADIN were radiographed, and its skull was prepared for closer examination.

### Virus Characterization/Isolation

In an attempt to reactivate and isolate latent FIV by mixed lymphocyte reaction (MLR), lynx peripheral blood mononuclear cells (PBMC) purified from heparin-anticoagulated whole blood were co-cultivated with PBMC from either uninfected lynx or specified pathogen-free domestic cats. Total nucleic acids (TNA) samples isolated from cell culture supernatants collected at different time points were tested by FIV RT-qPCR (28, 37). A whole genomic sequencing approach by next generation sequencing (NGS) was also performed (38). Reverse transcriptase (RT) activity was assessed in supernatants of the co-cultures using the product enhanced reverse transcriptase (PERT) assay (39, 40). Detailed methods are described in Supplementary Material 2.

## Results

### Case Description

Three free-ranging Eurasian lynx caught in the compartment Jura North were tested FIV-seropositive by WB but resulted negative by FIV-Provirus qPCR. They were subsequently monitored in quarantine enclosures for 36 days (ADIN), 40 days (NAIA), and 32 days (SENI) prior to euthanasia and necropsy. An overview of animal data, clinical findings, and results of pathogen screenings at different time points is given in Tables 2, 3. Details on abnormal blood values can be found in Supplementary Material 3.

**Table 3:** Pathogen screening at different time points of the three Eurasian lynx (*Lynx lynx*) seropositive for feline immunodeficiency virus by Western blot: 1st field capture (C1), 2nd field capture (C2), during quarantine (Q), and before euthanasia (E).

**Sample**	**Pathogens**	**ADIN**				**NAIA**				**SENI**			
		**C1**	**C2**	**Q**	**E**	**C1**	**C2**	**Q**	**E**	**C1**	**C2**	**Q**	**E**
Serum	Feline immunodeficiency virus (Ab, WB)	neg	**pos**		**pos**	p24	**pos**	**pos**	p24	Neg	**pos**	p24	p24
	Feline leukemia virus p15E (Ab)	neg	neg			neg	neg			neg	neg		
	Feline leukemia virus p27 antigen		neg		neg		neg				neg		
	Feline herpesvirus (Ab)	neg	neg			neg	neg				neg		
Whole EDTA-blood	Feline immunodeficiency provirus		neg				neg				neg		
	Feline leukemia provirus		neg				neg	neg			neg		
	Feline herpesvirus		neg				neg	neg			neg		
	Canine distemper virus		neg				neg	neg			neg		
	*Mycoplasma haemofelis*							neg				neg	
	*Candidatus* Mycoplasma haemominutum							**pos**				neg	
	*Candidatus* Mycoplasma turicensis							**pos**				neg	
	*Anaplasma phagocytophilum*							neg				neg	
	*Rickettsia species*							neg			neg	neg	
Oropharyngeal swabs	Feline calicivirus		neg				neg		neg		neg	neg	neg
	Feline herpesvirus		neg				neg		neg		neg	**pos**	neg
	Canine distemper virus		neg				neg		neg		neg	neg	neg
Conjunctival swabs	Feline calicivirus		neg				neg	neg			neg	neg	neg
	Feline herpesvirus		neg				neg	neg	neg		neg	neg	neg
	Canine distemper virus							neg					
	*Chlamydia felis*							**pos**	neg				neg
	*Mycoplasma felis*							neg					neg
Nasal swabs	*Chlamydia felis*											neg	
	*Mycoplasma felis*											**pos**	neg
Fecal swabs	Feline coronavirus		neg				neg		neg		neg		neg
	Feline parvovirus		neg				neg		neg		neg		neg

*Ab, antibodies; WB, Western blot; neg, negative; pos, positive*.

*Empty cells: not tested*.

**Figure 2:**
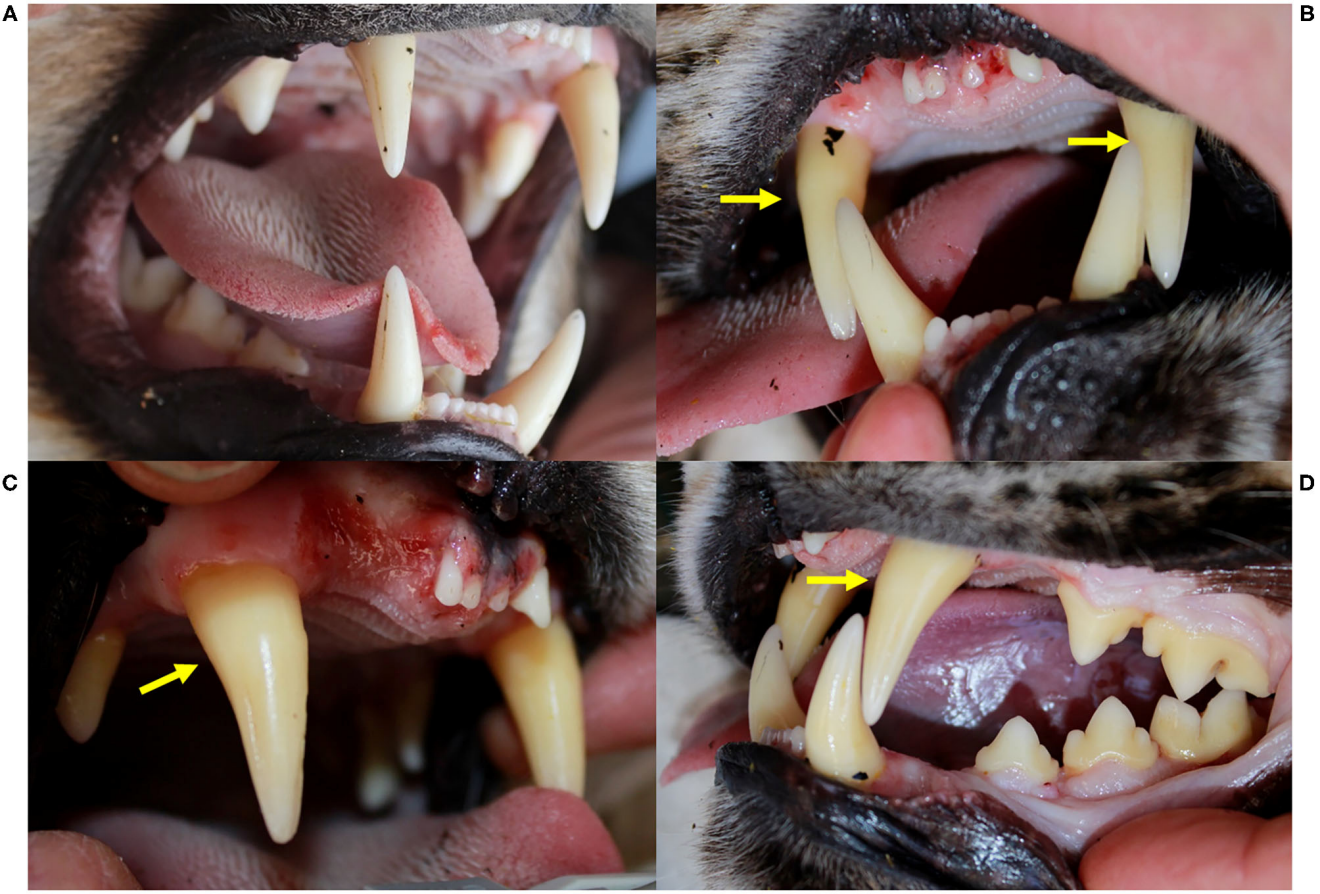
Oral health status, Eurasian lynx (*Lynx lynx*), ADIN; **(A)** Left fronto-lateral view of the opened mouth, 1st capture (2013, FIV-negative by Western blot): complete permanent dentition, intact gingiva. **(B)** Frontal view, **(C)** Left fronto-lateral view, and **(D)** Left lateral view of the opened mouth, 2nd capture (2016, FIV-positive by Western blot): missing upper incisors (103, 202) with multifocal spots of mild to severe gingivitis without obvious extraction wounds, intact mandibular incisors and intact enamel.

At their first field capture, ADIN (young adult in 2013) and SENI (8–9 months old in 2016) were in good body condition and had a complete and intact dentition with a healthy oral mucosa (Figure 2). Except for hypotrichosis on the ventral abdomen of SENI, they were both apparently healthy and were released on site with a radio-collar. At the time, they were FIV-seronegative by WB.

ADIN was re-captured in the field mid-March 2016. He was caught on a day that was not foreseen for captures, resulting in an abnormally long waiting time in the box trap during a mild sunny day. Reduced skin turgor suggested mild dehydration. His mandibular dentition was intact, while he had lost multiple maxillar incisors and had severe multifocal gingivitis in this area (Figure 2). At least part of the lesions were likely acute self-inflicted injuries that occurred while waiting in the box trap. He was tested negative for FIV with the POC test, transferred to a quarantine station in Austria, and found to be FIV-seropositive by both ELISA and WB. During quarantine, he developed anorexia and lethargy. Despite uncertainty regarding the etiology of these unspecific behavioral changes, in view of the health risk represented by a FIV-seropositive animal in case of a release into the wild (whether the source or the destination population) and of animal welfare implications of a prolonged stay in captivity, he was euthanized in agreement with all responsible authorities. He tested FIV-seropositive again by WB at the time of euthanasia.

**Figure 3:**
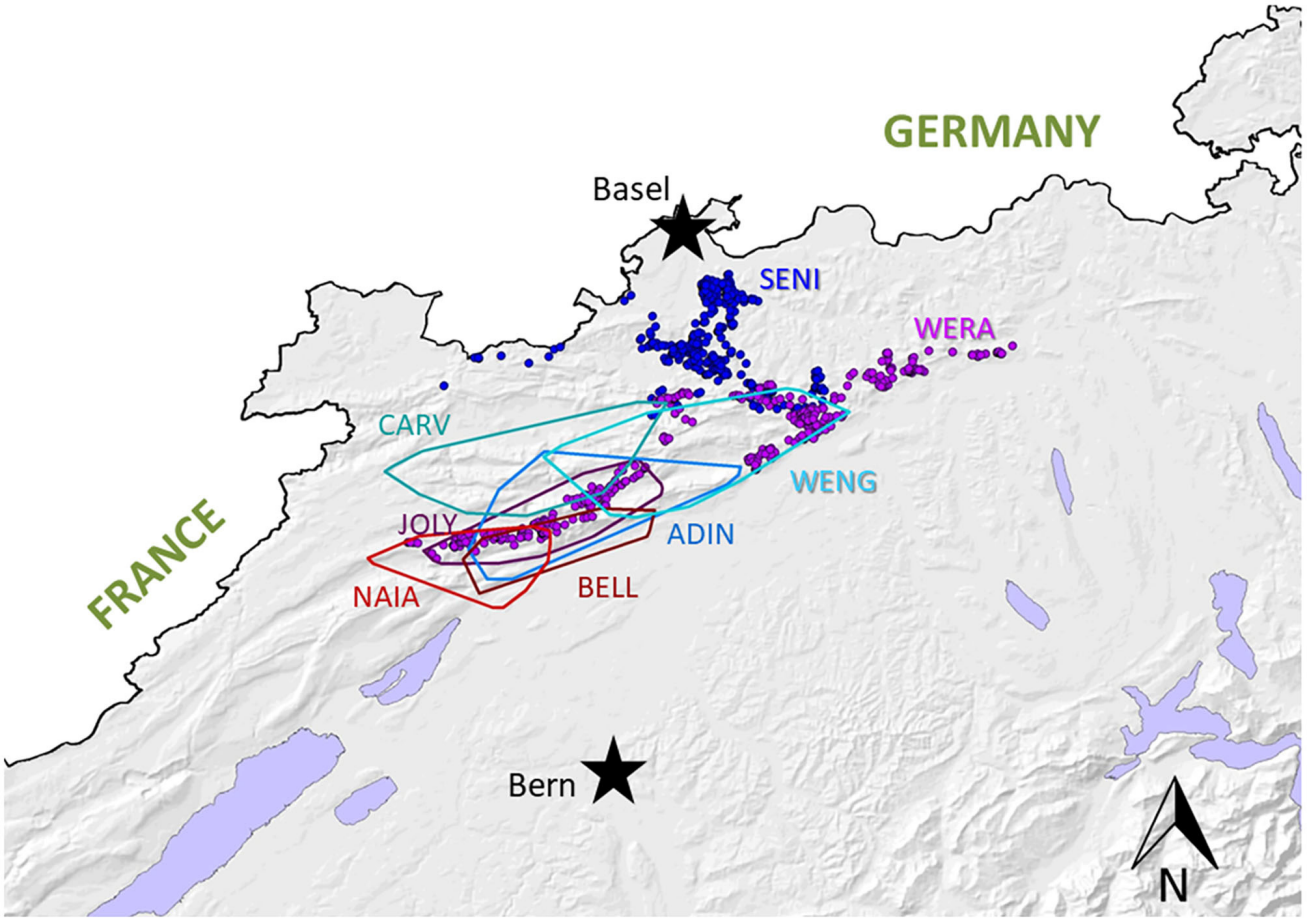
Map depicting home-ranges of the six radiomarked adult lynx in the compartment Jura North, including the males CARV (known since 2010 thanks to camera-trap monitoring, first collared in 2013), ADIN (known since 2012, collared in 2013), and WENG (known since 2015, collared in 2016), and the females JOLY and NAIA (both known since 2012 and collared in 2016), and BELL (known since 2013, collared in 2016). Of the two subadult lynx SENI (collared further north in 2016) and WERA (known since 2015, collared in 2017), the individual locations are presented as they did not yet have established home-ranges.

**Figure 4:**
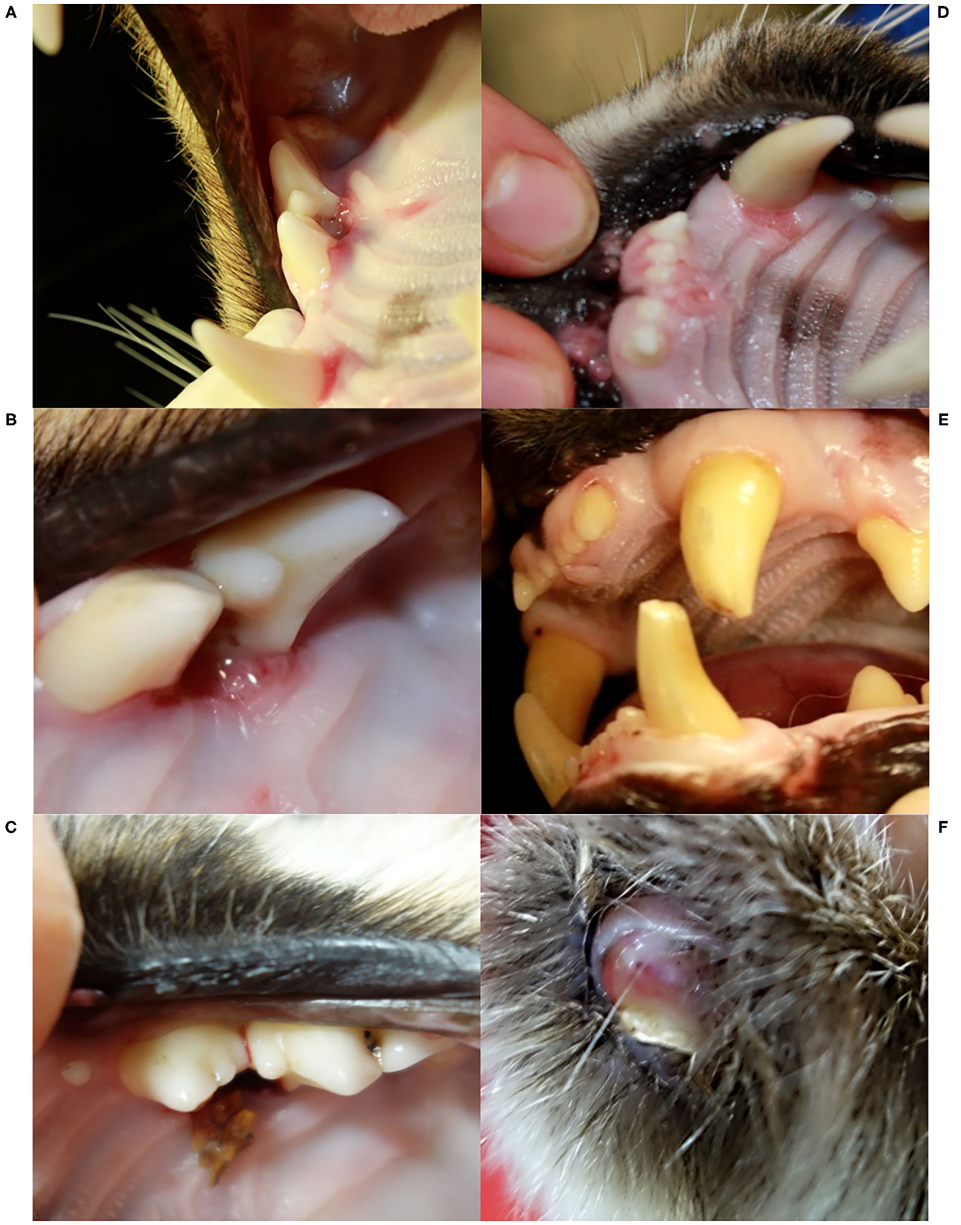
Oral cavity **(A–E)** and claw **(F)**, Eurasian lynx (*Lynx lynx*), examination during quarantine; **(A)** and **(B)** SENI: marginal gingivitis around left maxillar teeth; **(C)** SENI: socket formation and intralesional wood fragment between the premolars; **(D)** SENI: multifocal palatal erosions at the basis of the incisors and left canine; **(E)** Teeth, NAIA: three fractured canines with exposure of the pulp chamber of the two left canines; **(F)** Claw, SENI: Worn down claw with inflammation of the surrounding soft tissue.

**Figure 5:**
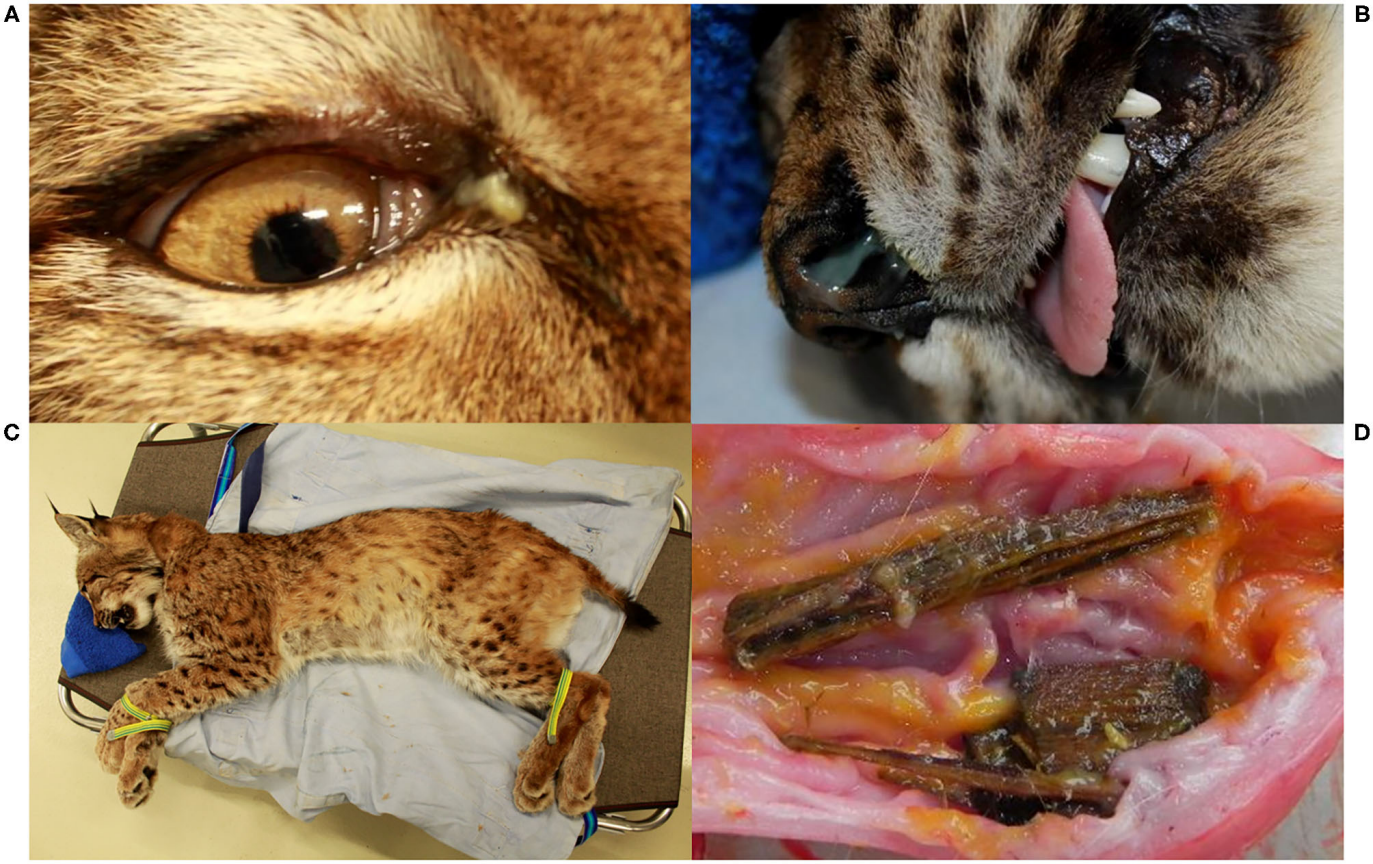
Various body parts, Eurasian lynx (*Lynx lynx*), examination during quarantine; **(A)** Right eye, NAIA, and **(B)** Nose, SENI: mild purulent nasal discharge. **(C)** Coat, SENI: Hypotrichosis, mainly visible on the belly, caudal thigh and tail; **(D)** Opened stomach, NAIA: pieces of wood embedded in the mucosa and partly covered by yellowish mucus.

A few weeks later, NAIA (young adult) was caught for the first time in the field and radio-marked in ADIN's territory (Figure 3), meaning the two may have previously mated. She appeared emaciated despite a weight within the normal range (Table 2) (41), had an already healed mating bite lesion on the neck, and abdominal palpation as well as teat appearance suggested pregnancy. She tested negative for FIV by POC test and ELISA and showed only an unspecific p24 band reaction in the FIV WB. She was released on site with a radio-collar and subsequently displayed a normal spatial behavior but did not give birth to kittens (no stationary behavior during the lynx birth period). When she was re-captured the following year (February 2017, i.e., ~10 months later) and brought to quarantine, she was in good body condition and again FIV-negative by POC test but FIV-seropositive by WB, which excluded a release into the wild. Four weeks later, she was anesthetized at the quarantine station for repeated sample collection and clinical examination. She had lost condition, presented with fractured canines (left with pulp exposure, right uncomplicated, Figure 4), and mild hypotrichosis of the tail and neck. She also had unilateral mild conjunctivitis with purulent ocular discharge (Figure 5) and tested positive for *C. felis* in the corresponding conjunctival swab. Blood pathogen screening revealed an infection with *Candidatus* Mycoplasma (*C*.M.) haemominutum and *C*.M. turicensis and she was still FIV-seropositive by WB (Table 3).

SENI was captured for the second time in the field as a subadult (2017) a week after NAIA, about 12 km away from ADIN's home range limit and 41 km south-west of his first capture site (in 2016) (Figure 3). He was probably on the search for a new territory. Like NAIA, he appeared emaciated despite a body weight within the normal range (Table 2) (41), with marked muscle atrophy of the hind legs, and presented with small healing wounds (with alopecia and crusts) on the right side of the chest and thigh, as well as marked tail hypotrichosis. He tested FIV-negative by POC test and was brought to quarantine. Like NAIA, he was subsequently found FIV-seropositive by WB, kept in prolonged captivity, and re-examined approximately 2 weeks after his arrival. At this time point, his body condition had slightly improved, but in addition to the tail, hypotrichosis affected the anogenital area, thighs, abdomen, and neck (Figure 5). Palpation suggested lymphadenomegaly of mandibular and popliteal lymph nodes. He showed mild hemorrhagic claw damages on the forefeet, a canine tip was fractured, and there was multifocal, focally mildly purulent gingivitis as well as erosions of the palate mucosa, mostly at the tooth neck (Figure 4). He tested positive for FHV in oropharyngeal swab and for *M. felis* in nasal swab. He showed only a p24 band in the WB (Table 3).

**Figure 6:**
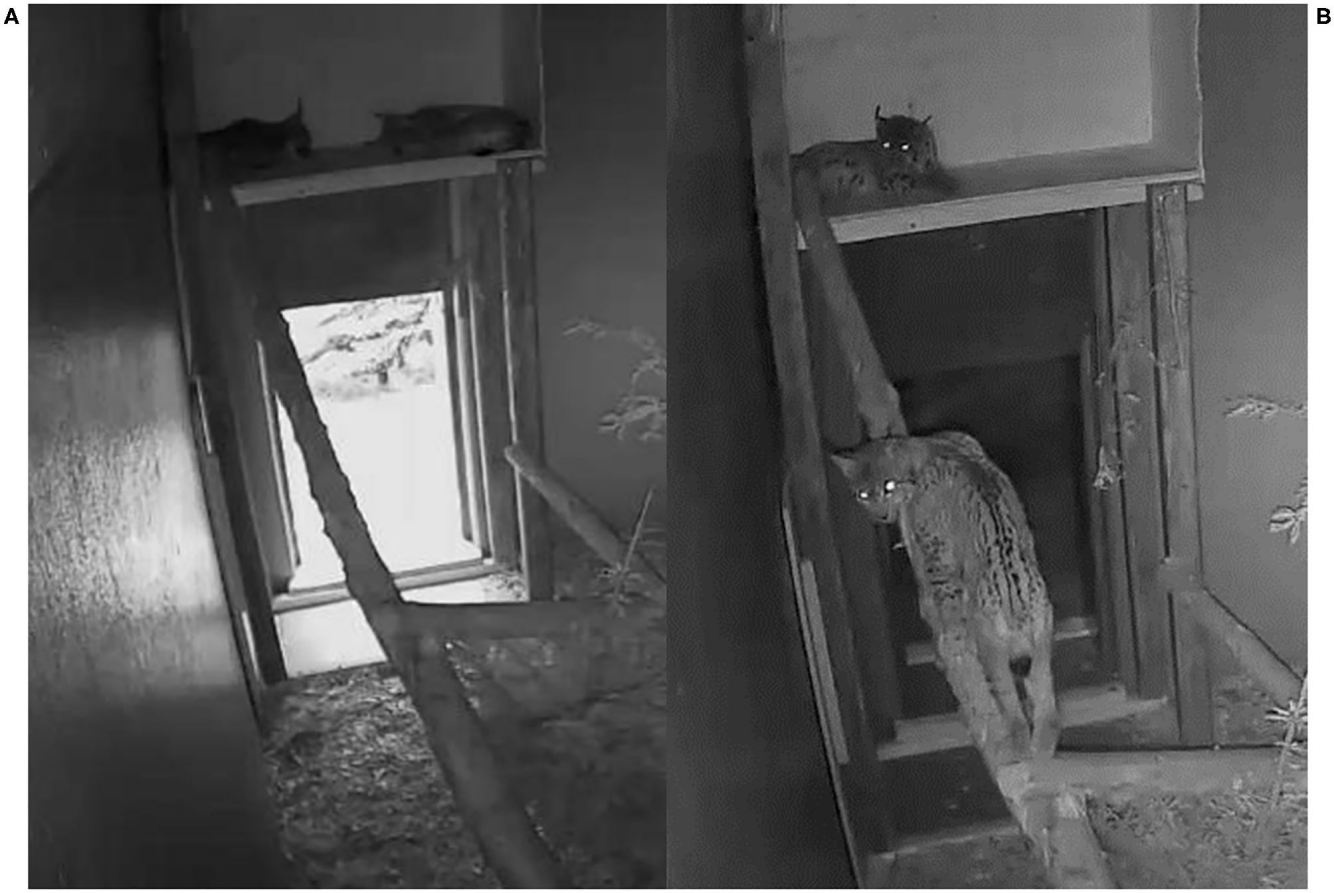
Behavior during quarantine as captured by video-monitoring, Eurasian lynx (*Lynx lynx*), NAIA and SENI; **(A)** Lethargy during daytime; **(B)** Moderate activity at night, SENI on the tree trunk in poor body condition.

Because only two enclosures were available for the on-going translocation project and there was interest in keeping these two FIV-seropositive lynx under close observation in captivity for a longer time period, SENI was moved to NAIA's enclosure. After an initial period of aggressive interactions, their relationship became more friendly and they even began to share the platform where they rested during the day. Their health status progressively deteriorated, including progressive anorexia and emaciation (Figure 6) together with behavioral abnormalities such as increasing lethargy and screams of distress at night. Activity and food intake of both animals progressively decreased during quarantine. Feeding duration continuously declined from 25 +/– 5 min at the beginning to 5 min at the end. SENI had watery diarrhea twice with negative coprology (McMaster method). Analyses of rectal swabs were negative for FCoV and FPV (Table 3). They were euthanized for animal welfare reasons.

Before euthanasia, NAIA and SENI underwent an additional clinical examination. NAIA appeared slightly emaciated and had crust-covered wounds on an elbow and the forehead. Tail hypotrichosis was unchanged, and there were no new oral or claw damage. By contrast, hypotrichosis had extended in SENI, affecting nearly the whole ventral body half, with skin hyperpigmentation and a small alopecic focus on the ventral aspect of the tail. He was emaciated with muscle atrophy, in particular of the hind legs, had severe claw damages (multiple claws worn down to their basis with inflammation of the surrounding skin (Figure 4), and palpation of superficial lymph nodes suggested lymphadenomegaly, in particular of the popliteal lymph nodes. The palatal erosions had healed but the focal gingivitis noticed earlier had developed into a periodontal pocket with intralesional foreign bodies (wood pieces, Figure 4). At the time of their euthanasia, NAIA and SENI showed again only a p24 reaction in the FIV WB. Analysis of NAIA's conjunctival swab was negative for *C. felis*, and SENI's oropharyngeal and nasal swab samples were negative for FHV and *M. felis*.

Hematology revealed normocytic, nonregenerative anemia in ADIN (at the time of euthanasia) and SENI (both at quarantine and pre-euthanasia examinations). SENI also had leucocytosis (due to neutrophilia and monocytosis) and elevated liver enzymes at the quarantine check. Blood chemistry revealed hypocalcemia in the three FIV-seropositive lynx at their second field capture, when they were found to be FIV-seropositive. NAIA had hyperproteinemia at her first field capture, when she was pregnant, and increased creatinine both at the quarantine and pre-euthanasia examinations. SENI and NAIA also had low cholesterol values.

**Figure 7:**
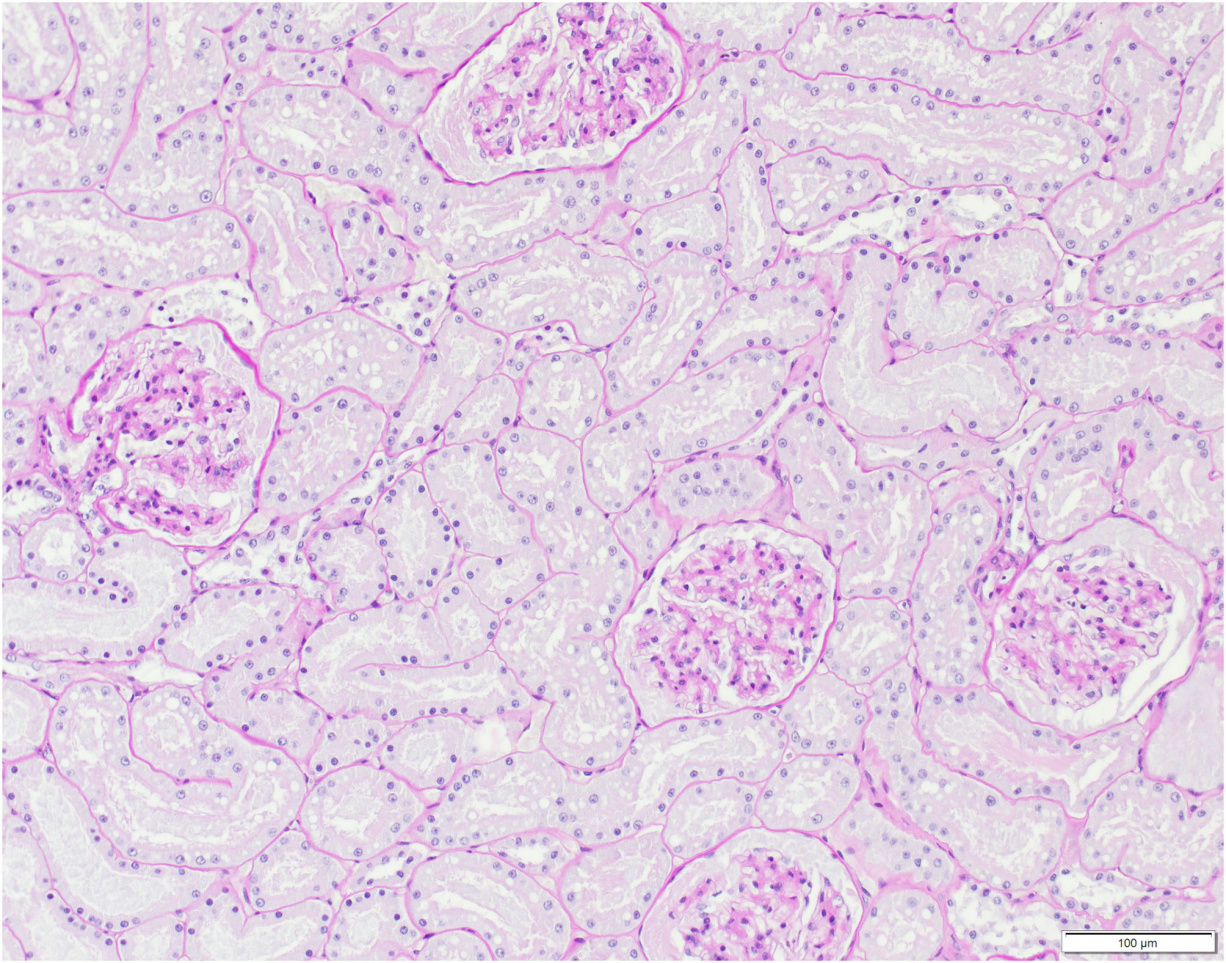
Histological kidney section, Eurasian lynx (*Lynx lynx*), SENI: Presence of eosinophilic material compatible with protein aggregates in glomeruli and tubules, with mild widening of the Bowman's capsule (mild, multifocal membranous glomerulopathy).

**Figure 8:**
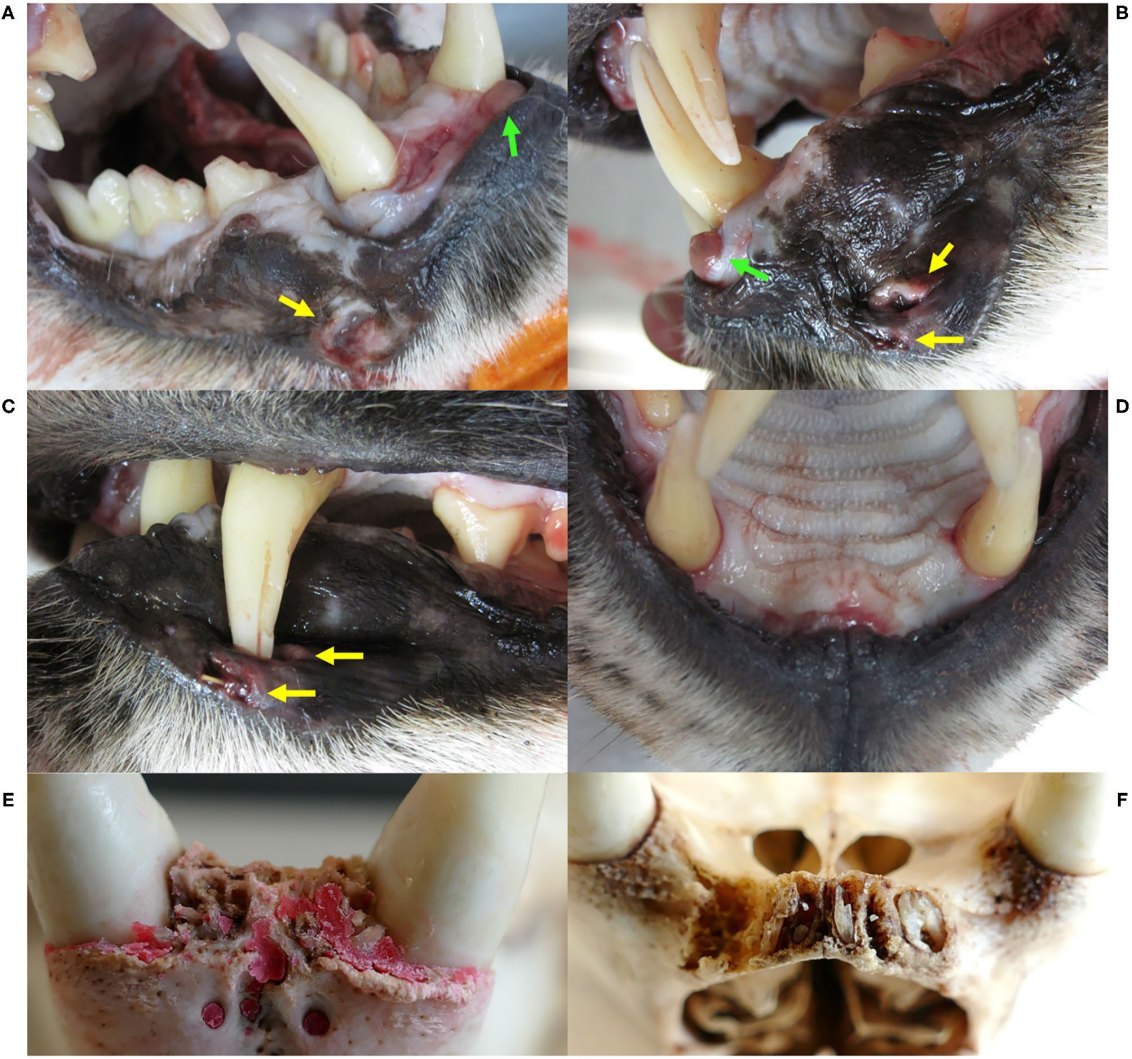
Dental and gingival lesions, Eurasian lynx *(Lynx lynx*), ADIN; **(A)** Right fronto-lateral view and **(B)** left lateral view of the opened mouth, necropsy: bilateral wound (yellow arrows) possibly caused by puncture of the maxillar canines, a tumorous tissue formation (green arrows), tooth abrasions of the right premolars and first mandibular molar, and enamel defects of the maxillar left canine. **(C)** Left lateral view of the closed mouth, necropsy, with closed mouth, showing lip perforation by the upper left canine aside from an open wound with intralesional foreign body. **(D)** Fronto-ventral view of the maxilla, necropsy: all incisors missing with focal gingivitis. **(E)** and **(F)** Ventral views of the frontal part of the mandibula and maxilla, respectively, prepared skull: all incisor crowns missing with and chronic osteomyelitis and root remnants in the maxillar alveoli. Red deposits on picture E are artifacts.

**Figure 9:**
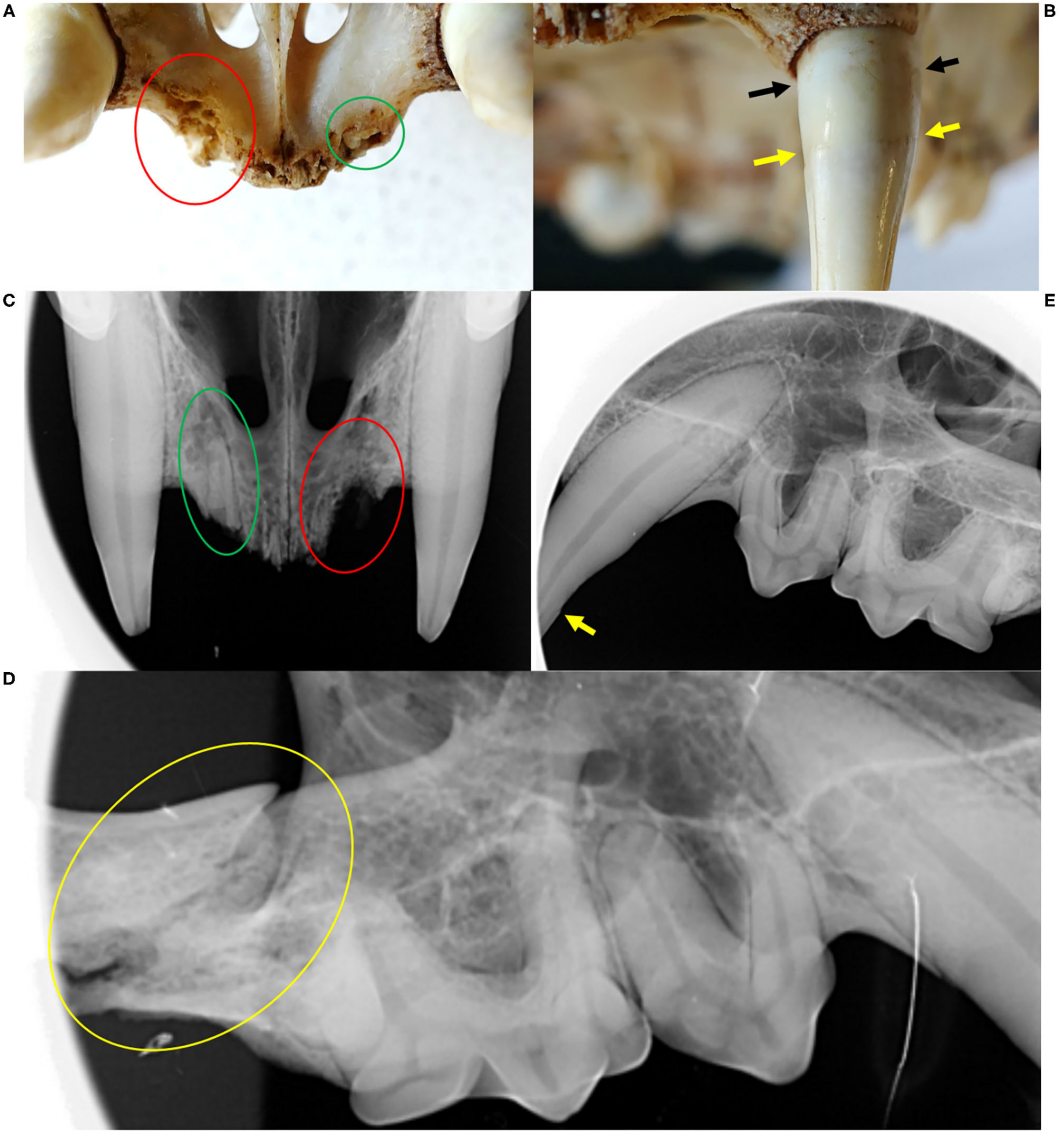
Dental and bone lesions, Eurasian lynx (*Lynx lynx*), ADIN; **(A)** Prepared skull and **(C)** Radiograph, fronto-ventral view of the maxilla: all incisors missing with chronic osteomyelitis and osteolysis (red circle); visible root remnants of incisor 203 (green circle). **(B)** Prepared skull, frontal view of the maxilla: two distinct lines (cemento-enamel junction: yellow arrow, and gingival line: black arrow); **(D)** Radiograph, left lateral view of the maxilla: contour of the canine root perfectly followed by the alveolar wall; **(E)** Right lateral view of the maxilla: fracture of the zygomatic arch (yellow circle).

Pathological observations are summarized in Table 4. In addition to disease signs noticed at clinical examinations, post-mortem investigations revealed absence of food in the stomach, ingested foreign bodies, membranous glomerulopathy (Figure 7) and lymphoid depletion (either lymph node or spleen) in the three lynx. ADIN had a mixture of straw and mushy digesta in the intestine, while SENI and NAIA had swallowed pieces of wood from the enclosure ground (size of approximately 2–5 x 1.5–2 x 0.5 cm) that were stuck in the pylorus, partly adherent to the gastric mucosa, and associated with chronic gastritis (moderate and ulcerative in NAIA, mild with fibrosis and duodenitis in SENI). Both had an empty small intestine and poorly filled large intestine that contained dry green mushy to fibrous content. SENI also had over 10 wood pieces and hairs clumped together right before the Ostium ileocaecale. In addition, SENI and NAIA had mild purulent bronchopneumonia. ADIN showed severe gingival and dental pathologies, including loss of all incisors, which were either broken or lost in association with chronic, severe periodontitis, bilateral perforations of the lower lips associated with the maxillar canine tips, with socket formation and open wound with intralesional foreign body (possibly straw). Although the cemento-enamel junction suggested tooth extrusion, this was not supported by radiological examination. There were also severe enamel defects, canine and premolar abrasion, and molar periodontitis. Furthermore, a fracture of the zygomatic arch was visible on a radiograph (Figures 8, 9).

**Table 4:** Pathological findings including macroscopical and histological diagnoses of the three *FIV-seropositive* Eurasian lynx (*Lynx lynx*). Empty cells: not detected.

**Pathologies^**a**^**	**Case 1 (ADIN)**	**Case 2 (NAIA)**	**Case 3 (SENI)**
Membranous glomerulopathy	++	+	+
Lymphoid depletion	+ lymph node	++ spleen	+ spleen
Meningitis		+	
Pneumonia		+	+
Hypotrichosis		+	++ with alopecia, hyperpigmentation, mite-like parasite
Myocarditis		+ with fibrosis	
Ingested foreign bodies	+ (no stomach or intestine samples for histology)	++ with moderate gastritis	+++ with mild gastritis and duodenitis
Tooth fractures	+++ with severe periodontitis, tooth loss, gingivitis and osteomyelitis	++	++ with periodontitis
Hepatic lipidosis	+++		
Adrenal hypertrophy	+++		
Emaciation		+	++

a *Graded as mild +, moderate ++, or severe +++*.

Additional post-mortem findings in ADIN were severe diffuse hepatic lipidosis, severe diffuse cortical adrenal hypertrophy, lymphadenomegaly (mesenteric lymph nodes), absence of mature spermatozoa, claw splits, and a few wounds on the forepaws (a purulent puncture wound and two focal ulcerations). Further noteworthy findings in NAIA included unspecific, mild chronic myocarditis with cardiomyocyte degeneration and fibrosis, and mild, subacute to chronic, lymphocytic meningitis with mild gliosis including satellitosis. Parasitic cysts morphologically compatible with *Hepatozoon* spp. without associated myocarditis, and superficial lice were observed in the examined tissue sections of SENI and subsequent observation with by stereomicroscopy of a frozen skin sample.

### FIV Serology on Lynx Samples

From 1993 to 2014, FIV serology had always been performed on lynx by ELISA (42). All of them (*n* = 108) had been either negative (<9%) or occasionally doubtful but confirmed FIV-negative by WB (p24 band only, if any). Considering the sensitivity issues newly encountered with the ELISA (14), starting from 2015 disease screening protocols were changed to WB for FIV serological testing.

When NAIA was tested FIV-negative by ELISA but FIV-seropositive by WB lynx in 2017, the question was raised as whether former ELISA negative results were reliable. Therefore, we retested by WB 123 archived serum samples from 84 free-ranging live lynx of both sexes (39 males, 45 females) and different age classes [27 juveniles incl. eight newborn kittens, i.e., approximately 4-week-old, 11 subadults and 46 adults; for age estimation see (23, 43)] from the three Swiss lynx populations (26 from the Jura, 50 from the Alps, eight from northeastern Switzerland) captured before trapping ADIN mid-March 2016, plus four lynx caught in April-May 2016, namely, NAIA at her first capture and the subadult female WERA in Jura North (Figure 3), an adult female in the Alps, and a subadult male in northeastern Switzerland). Each animal was tested at least once, with 29 individuals subsequently kept in provisory captivity (quarantine station or rehabilitation center) that were re-tested up to six times. These 84 lynx included SENI, NAIA, and ADIN at their first field capture. Additionally, our sample collection comprised samples of three zoo lynx kept in the same enclosure.

Of the 84 free-ranging lynx tested retrospectively, including 17 from Jura North (2003–2016), none was found FIV-seropositive. Twenty-seven lynx (32%) of both sexes, all age classes and originating from the three populations showed a possibly unspecific p24 band reaction when tested for the first time (Figure 1, Table 1). Of the 29 animals tested more than once, six “acquired” reactivity to the p24 band (five during captivity), and five lynx “lost” this band (two during captivity). None of the eight newborn kittens (from five females) were seropositive, but three (from the same female in the Alps) showed a p24 band reaction. The other lynx did not change status despite being tested up to four times over several months. Two of the three zoo lynx showed a p24 band reaction. Twenty-six animals that showed a p24 band or were found questionably positive by ELISA were further tested by FIV provirus qPCR and found negative.

From then on, the ELISA was no longer performed. Excluding NAIA and SENI, from February 2017 to April 2021, 40 more lynx were captured and tested for the first time by WB (24 males, 16 females; nine juveniles including two newborn kittens, six subadults and 25 adults; 22 from the Jura comprising 10 lynx from the compartment Jura North, 13 from the Alps, five from northeastern Switzerland). None of these 40 lynx was found FIV-seropositive, and 22 (55%) showed a p24 band reaction. The two kittens born from the female JOLY in Jura North (Figure 3) in June 2017 were negative. Four lynx tested twice with or without a p24 did not change status, and two lynx in provisory captivity “lost” and “acquired” a p24 band, respectively. Additionally, the male CARV and three adult females that had been first tested before 2017 were re-captured and tested a second time. All had remained negative, partly “losing” or “acquiring” a p24 band in the meantime.

In total, we tested 124 free-ranging lynx sampled from 2001 to 2021 for FIV antibodies by WB at their first capture, including 47 lynx from the Jura (of which 26 were caught in the compartment Jura North), all with a negative result, with or without p24 reaction (apparent prevalence of 0, 95% confidence interval 0.0–0.03%; epitools.ausvet.com.au). However, three lynx with geographically close territories (ADIN, NAIA, and SENI) were FIV-seropositive (3/127, 2.4, 0.8–6.7%) at their second capture in 2016–2017. Lynx with a p24 reaction were found during the whole study period, in animals of both sexes in all age classes and geographically scattered across the three lynx populations (Figure 1).

### Disease Management Strategy

**Figure 10:**
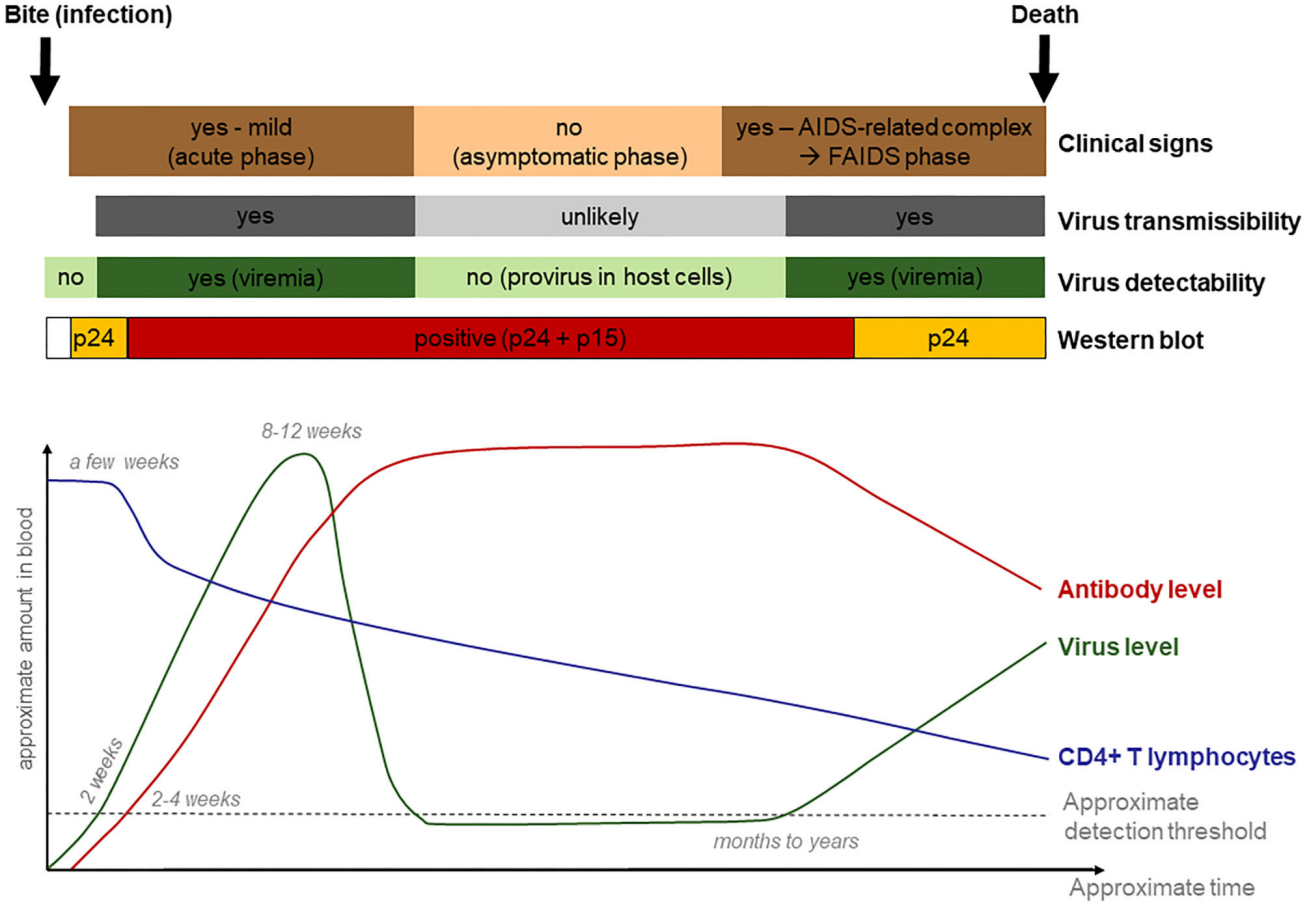
Tentative graphical representation of feline immunodeficiency virus infection dynamics, with clinical manifestations and diagnostic possibilities.

After the second and third FIV-seropositive lynx cases were recognized, the on-going lynx translocation programs and necessity to exclude possibly FIV-seropositive lynx requested the development of a disease management strategy that went beyond the exclusion of FIV-seropositive lynx as originally planned (23). This need arose from the risk of a negative WB result during the first phase of FIV infection (3). For this purpose, relevant information was gathered from the literature that may help define criteria to help recognize and exclude lynx infected with FIV (Supplementary Material 1). Infection dynamics of FIV and associated diagnostic challenges, as known from domestic cats, are tentatively summarized in Figure 10.

Based on the available information and lynx-specific situation, there were serious constraints to consider when developing the scheme. First, diagnostic problems inherent in FIV infection dynamics included the low probability to detect antibodies in the first weeks post infection. Second, during the initial FIV infection phase, animals may show only a p24 band on WB strips. However, a p24 band may also result from an unspecific cross-reaction with another unrecognized viral infection. Ideally, lynx with an uncertain test result (p24 band) would be kept in quarantine for a few months for re-testing, like it is recommended for domestic cats (3). However, this procedure was not considered an option for lynx due to animal welfare reasons. Indeed, lynx born in the wild are very stressed when kept in an enclosure and tend to injure themselves due to nervous behavior and escape attempts (23). Also, females caught during the mating season, especially those with a neck bite wound (whether recent or healed), may be pregnant. Considering that the mating season (and also the capture season) lasts from mid-February to mid-April (44) and lynx pregnancy has a duration of 70–75 days (45), keeping females captured late in the season for more than a month in captivity would bear the risk that they may give birth before transport to the destination site, or be released just before birth without getting any opportunity to first adapt to the new environment. An additional difficulty is that FIV infection is usually accompanied by unspecific or absence of clinical signs; thus, clinical appearance does usually not help to support the diagnostic process.

Third, transmission of FIV occurs mainly by bites. Intraspecific lynx biting is most likely to occur during the mating season, as occasional fights between males are more common during this period (46), and lynx males cause a severe mating bite to females during copulation (47). Because the best lynx capture period corresponds to the mating season (23), the likelihood is high that a female would present a neck wound at capture, i.e., such females with a p24 band may either be uninfected (unspecific reaction) or in an early phase of FIV infection with no possibility to distinguish between the two. Last but not least, political pressure on wildlife managers resulting from long-lasting local conflicts with hunters (48, 49) excluded the option of repatriating lynx with a p24 band to their original capture site and required a pragmatic solution, even if this was likely the safest procedure in term of disease risk.

**Figure 11:**
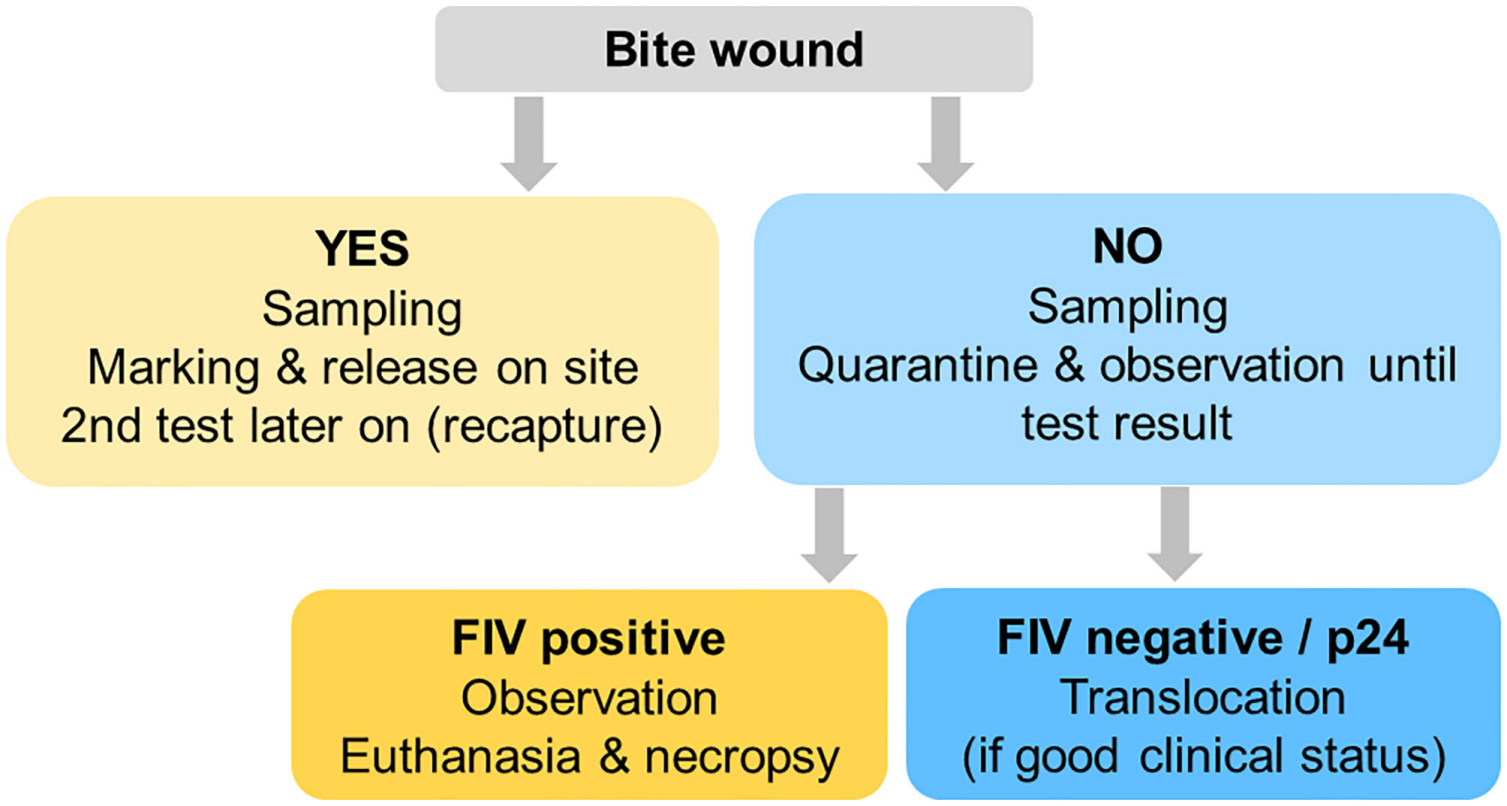
Decision scheme 2017–2020 for lynx captured for translocation purposes, aiming at minimizing the disease risk potentially associated with a feline immunodeficiency virus infection without jeopardizing conservation efforts.

Taking all above-mentioned challenges and the results of the retrospective screening for FIV by WB together, we developed a decision scheme for case management aiming at minimizing FIV-related health risks (for both the destination and the source populations) while avoiding jeopardizing the ongoing conservation efforts (lynx were urgently required for reintroductions elsewhere, the political will to support translocations was given, and necessary funding was allocated for just a few years). The scheme was meant for the compartment Jura North only. It required exclusion of all lynx presenting with a recent (fresh or healing) wound, to avoid removing a lynx from its territory that may be in the initial infection phase. Nevertheless, such lynx would be blood sampled and tested for FIV and may be re-captured or shot later if found FIV-seropositive (Figure 11). In agreement with the project partners in the destination areas, it was decided that lynx without wounds brought to quarantine with an apparently good health status would be translocated, whether or not they would show a p24 band in the WB. FIV-seropositive lynx would be euthanized. In domestic cats, positive animals shall be confined indoors to prevent infection of other cats (50) but this was not an option for wild-born lynx.

Four females were subsequently captured from Jura North that presented with a neck bite and were therefore released on site. All were FIV-seronegative.

### Virus Identification

Different approaches were chosen to identify and characterize FIV in these animals. Three different qPCRs (one in-house and two commercial) and three nested/semi-nested conventional PCR were performed to detect FIV proviral DNA, but results were always negative. A faint PCR band with the correct amplicon length was detected in the sample of ADIN by semi-nested PCR (35, 36), but sequencing did not reveal any FIV compatible sequences. Furthermore, FIV RT-qPCR testing of TNA isolated from cell culture supernatant of the PBMC co-cultures also resulted negative and no FIV reads were detected in the NGS approach. On the contrary, a very weak reverse transcription (RT) activity was detected in the supernatant of the co-culture of ADIN PBMC with a SPF cat (39 nU/ml PERT) and in the co-culture of NAIA PBMC with an uninfected lynx (25 nU/ml PERT). The co-culture of a positive control sample (latent FIV-infected domestic cat) was positive by FIV RT-qPCR (CT-value 20) and showed a very high RT activity (4.5 x 10^7^ nU/ml PERT). Negative co-culture controls were negative both by FIV RT-qPCR and PERT assay.

## Discussion

For the first time in nearly 20 years of health monitoring in live free-ranging lynx in Switzerland, we were confronted with the challenge of managing animals that were possibly infected by FIV in a context of international translocations. Previous experiences included over 300 clinical examinations of more than 150 lynx of different age classes and both sexes as well as thorough health assessments and quarantine observations of multiple animals for four different translocation projects and for rehabilitation, and even more animals have been collared and monitored by radiotelemetry. Similarly, complementary health monitoring of dead lynx initiated in the 1970s (23) has concerned several hundred animals to date. In other lynx species, FIV has been detected in bobcats in the United States but potentially associated disease signs have not been reported (51). Absence of exposure was documented in Iberian lynx in Spain (20), and data on Canada lynx (*Lynx canadensis*) are not available.

Clinical signs and pathological changes observed in the three FIV-seropositive lynx were in agreement with reports from FIV-seropositive domestic cats. Since FIV can induce immunosuppression, clinical signs in infected animals result mainly from secondary infections and are very variable. Lethargy, anorexia, diarrhea, poor oral health, poor hair coat, lymphadenopathies, nephropathies, and opportunistic infections are typically observed in FIV-infected cats (Supplementary Material 1). Yet, capture, transport, and especially captivity are a source of intense stress for wild-born lynx, bearing the risk of self-inflicted injuries such as claw, oral soft, and hard tissue injuries during escape attempts, as well as stress-induced immunosuppression (23, 52, 53).

The three lynx developed anorexia and lost weight during quarantine, although their weight was within the normal range for their respective age and sex category at all time points (41), even after taking their body size in consideration. Weight differences may be attributed to a decreasing amount of stomach content, but SENI already looked in reduced body condition before quarantine (at his second field capture), with visible muscular atrophy of the hindlegs. NAIA was also considered in suboptimal condition at all time points except right before quarantine (second field capture). This discrepancy between clinical evaluation and measured weight is unclear but reduced food intake during quarantine was doubtless as it could be objectively assessed based on carcass consumption. Furthermore, suboptimal condition in SENI and NAIA was obvious on video images (Figure 6) and confirmed at necropsy, although at post-mortem examination their nutrition state was much better than expected. Anorexia and emaciation is often observed in FIV-infected domestic cats but typically induced by severe stomatitis (3, 54). Oral health was compromised in the three lynx and worst in ADIN, which lost weight over time but was never considered emaciated. Nevertheless, he was diagnosed with hepatic lipidosis, which is a potentially fatal syndrome in domestic cat that typically results from an illness-induced catabolic state (55). Wasting and poor muscle condition is also common in FIV-positive African lions (*Panthera leo*) as well as humans and non-human primates infected with pathogenic lentiviruses (6). However, anorexia in ADIN may have resulted from stress and inadequate food. SENI and NAIA had ingested large wood pieces. While captive lynx occasionally bite wood structures and may ingest wood splits, it usually does not cause major problems and the material in the enclosures of NAIA and SENI was the same as for zoo lynx. As there was no accumulation of food higher in the digestive tract, it suggests that it had occurred rather shortly before euthanasia.

Mainly SENI but also NAIA had skin abnormalities. Chronic skin changes are common in FIV-seropositive cats (56) and poor hair coat was observed in a large proportion of FIV-seropositive compared to FIV-negative lions (6). However, transient skin and fur changes such as healed or infected skin wounds, tick (*Ixodes sp*.) and ear mite (*Otodectes cynotis*) infestations, as well as tail hypotrichosis of undetermined origin have previously been observed in numerous FIV-negative Swiss lynx. The fact that NAIA developed fur changes after becoming in contact with SENI indicates that it was a transmissible fur condition, which is in agreement with the lice found in SENI skin samples. Massive lice infestations with extended hypotrichosis have rarely occurred in free-ranging Swiss lynx and only in very stressed juveniles but lice were well visible in the fur. Immunosuppression, whether induced by a virus or stress, may have contributed to increasing lesion severity in SENI (52, 53, 57). Another unusual observation in the three lynx was mild reduced turgor noticed on several occasions suggesting potential minimal dehydration, which may have been linked to capture conditions in ADIN. Interestingly, poor coat and dehydration were noticed more frequently in FIV-seropositive than FIV-negative lions (6). However, hematological values did not indicate dehydration in the lynx.

SENI developed purulent nasal discharge during captivity, which may have been caused by FHV infection (58, 59), as per detection in an oropharyngeal swab. This herpesvirus infection typically starts in the nose, where it affects epithelial cells before spreading to the oropharynx, lids, and upper respiratory tract (59). An infection with FHV had not been previously diagnosed in Swiss lynx but only a few lynx have been tested with swabs so far and former serological investigations had shown that the virus circulates in the Swiss lynx population (23), making uncertain the relationship between this finding and FIV, unless immunosuppression had favored the development of clinical signs. Both NAIA and SENI were FHV qPCR negative when they arrived at the station, meaning no viral excretion was detected at the time. In domestic cats, FHV is transmitted in situations of close contacts, while droplet infections over larger distances seem to be rare. Thus, stress (or FIV) may have caused a reactivation of a latent infection in SENI (59). However, this was not supported by seronegativity to FHV immediately before transport to the quarantine station (second field capture). Furthermore, clinical signs had disappeared by the time of euthanasia, and such transient signs may occur and remain unnoticed in many free-ranging lynx. *Mycoplasma felis* was detected in SENI's nasal swab but this pathogen is known to mainly cause conjunctivitis (60) and its contribution to the observed rhinitis is unlikely. At the time of euthanasia, SENI had mild dried unilateral purulent ocular secretion. He was negative for all pathogens tested in conjunctival and oropharyngeal swabs including FHV and *M. felis*, meaning he cleared infection without treatment. Given the parallel occurrence of gingiva lesions in SENI, we considered the potential involvement of FCV (59) but this virus was not detected at any of the three testing time points, and NAIA and ADIN were also tested negative (Table 3).

NAIA showed unilateral conjunctivitis with fresh purulent ocular secretion during quarantine and tested positive for *C. felis*, which can cause ocular disease in domestic cats (60). A severe clinical case was formerly documented in a FIV-negative lynx (61), raising the question as why signs were only mild and transient if NAIA was immunosuppressed. NAIA was also found positive to *C*.M. haemominutum and *C*.M. turicensis in blood but such infections have been formerly documented in both Eurasian and Iberian lynx (*Lynx pardinus*), in which their pathogenicity is questionable (62–64). Similarly to SENI, NAIA cleared *C. felis* infection without treatment within 2 weeks.

Based on observations of free-ranging Swiss lynx by the authors, emaciation—even if only mild—in pregnant females and miscarriage are unusual. Although a few pregnant females did not give birth to kittens after translocation (23), we had not previously made observations suggestive of abortion of any marked free-ranging female lynx diagnosed as pregnant and released on site. Similarly, abnormal behavior such as displayed by NAIA and SENI during quarantine, diarrhea episodes of undetermined origin, increasing anorexia, ingestion of foreign material larger than small wood splits, and severe dental and gingival damages as developed by ADIN had not been previously observed in quarantined lynx kept in the same or similar conditions as NAIA and SENI, even when captivity duration extended over several weeks or when animals seemed very stressed. In the past, healthy lynx had refused food during up to 3–4 days after arrival at the quarantine station and a few had lost weight in very stressful situations such as repeated human disturbance but decreasing appetite had not occurred. ADIN, however, was housed in very inadequate conditions for a wild-born lynx. Furthermore, purulent bronchopneumonia as observed in NAIA and SENI is not a frequent post-mortem finding in Swiss lynx and may hint at an opportunistic infection in immunosuppressed individuals. A difference with other lynx was that NAIA and SENI shared the same enclosure. In a former translocation of an adult lynx pair during the mating season, the two lynx, which were kept outdoor in provisory captivity at the destination site before release, had broken the fence between them to stay together. Thus, keeping two lynx of different sexes in the same enclosure at this time of the year was not expected to be a major issue. However, SENI was only subadult and initial interactions between the two lynx were tense with occasional aggressive behavior, and the situation may have caused more stress than expected.

ADIN showed dramatic oral health degradation, progressively losing all his incisors, developing severe periodontitis with alveolar osteomyelitis, and enamel defects. Brittle incisor remnants suggested that breakage may have been favored by poor dental health such as tooth resorptions (TR). Chronic gingivitis and periodontitis are commonly observed in FIV-infected cats and are important etiological factors of TR. An association between FIV infection and TR was indeed demonstrated in an experiment with domestic cats (65). Poor oral health is also the most striking observation in free-ranging FIV-infected African lions (6). However, former studies suggested that dental fractures and periodontitis are frequent, while TR lesions are uncommon in free-ranging wild felids, including the Eurasian lynx (66–70). ADIN had been housed in particularly stressful conditions without video-surveillance system. Therefore, it is possible that he had injured himself by repeated biting on metal bars of the enclosure during the night. It is typical for quarantined lynx to remain apparently quiet during the day and to “decompensate” at night. The origin of the zygomatic arch fracture is unclear and may have occurred post-mortem during transport to the pathology laboratory, as no corresponding lesions were noticed in soft tissues at necropsy. Alternatively, it would indicate a severe trauma during captivity. Lip perforations likely occurred due to swollen tissues, as may have developed following repeated biting on hard structures in the enclosure. The original suspicion of canine extrusion was not supported by radiological examination. In lynx older than a year, the cemento-enamel junction of canine teeth is typically visible and relatively far from the tooth collar (43). This finding in ADIN is therefore not unusual for a lynx of his age. In domestic cats, gingival recession is typically associated with periodontitis (71). Thus, visible cemento-enamel junction at an increasing distance from the gingival margin over time, which seemingly corresponds to further tooth eruption or extrusion, is likely rather due to chronic gingival retraction. Although lynx from Switzerland were found to have a fairly good oral health for free-ranging wild felids, incisor loss, canine fractures, and periodontal disease are common (43, 67). Chronic gingival lesions are likely an issue also in other wild felids, as it was shown that measurements of gum-line recession can be used to age pumas (*Puma concolor*) (72). NAIA also fractured a few teeth during quarantine. While tooth fractures had occasionally occurred in stressed lynx caught in grid box traps or housed in poorly structured enclosures, they had never affected multiple teeth like in ADIN and NAIA.

At histology, the three FIV-seropositive lynx showed membranous glomerulopathy, in agreement with findings in FIV-positive lions (6). Though lesions may vary, renal involvement is frequent in FIV-infected cats, similarly to HIV-infected humans (73, 74) where, due to a type III hypersensitivity reaction, soluble immune complexes deposit in blood vessel walls causing inflammation and tissue damage (75). In contrast to Iberian lynx, which often present with membranous glomerulonephritis of immune origin in absence of FIV, FCoV, FCV, and FeLV infection (76), glomerulopathies are infrequent in Swiss Eurasian lynx. The three FIV-seropositive lynx also showed mild to moderate lymphoid depletion, in either lymph node or spleen. Lymphoid depletion is an unspecific reaction that can develop in case of viral infections such as canine distemper (77) but it is a hallmark pathology of immunodeficiency virus infections of humans, macaques (*Macaca mulatta*), and domestic cats, and it was also observed in FIV-positive lions (6).

During quarantine, ADIN, NAIA, and SENI displayed increasing lethargy. Although an alternance of inactivity and escape attempts is typical in free-ranging lynx kept in provisory captivity, lethargy as observed in these three individuals and screaming had not been previously observed in quarantined lynx. Both HIV and FIV enter the brain in the acute disease stage and can lead to neuropathies and unspecific behavioral disorders. Experimentally FIV-infected cats developed meningitis and inflammation of choroid plexus and cerebral white matter (78) and captive lions displayed periodic “star-gazing” behavior with a lack of response to stimuli, dysphagia, abnormal gait, ataxia, and lethargy, with various degrees and types of histological lesions in the central nervous system, including lymphocytic meningitis (79). NAIA had lymphocytic meningitis but no cerebral lesions and SENI did not show any lesion in brain samples (no data available for ADIN). Furthermore, screaming was observed in a highly stressed orphan kept in isolation and may have been a sign of distress, and not all lynx in quarantine have been monitored by video-surveillance.

Decreased hematocrit was observed in ADIN at the time of euthanasia (together with low hemoglobin value) and in SENI (both during quarantine and at euthanasia), though pale mucous membranes suggestive of anemia were not observed at clinical and post-mortem examination, and ADIN already showed hematological changes at his second capture. In FIV-infected domestic cats, anemia is not the main clinicopathological change but it may be present (80). These parameters were partly changed in ADIN and SENI, while other changes reported in domestic cats were not observed. Elevated serum total protein can occur in FIV-infected cats especially in advanced stages of FIV-infection (81, 82) but were not observed in the FIV-seropositive lynx, apart from transient hyperproteinemia in NAIA. SENI had elevated leucocytes with neutrophilia in quarantine and at euthanasia. This may have been related to the transient infections noticed during quarantine but is not in agreement with immunodeficiency. In domestic cats, CD4+ T lymphocytes are progressively replaced by CD8+ cells but CD4+ cells show a very rapid decrease in late diseases stages, during which also leukopenia (neutropenia) is common, though not always observed (56, 81, 82). NAIA had elevated creatinine during quarantine and at euthanasia, which may have been related to renal lesions (78), but these were worst in ADIN, which had a normal creatinine level. SENI also had elevated aspartate-aminotransferase (ASAT) and alanine-aminotransferase (ALAT) but like the few other changes in hematological and serum chemistry parameters of the FIV-seropositive lynx, the origin of these abnormalities is unclear. Interestingly, decreased values for hematocrit and hemoglobin, and elevated values for total protein, creatinine, ASAT, and ALAT, were also observed in infected lions (6). Clinicopathologic abnormalities may occur in FIV-infected cats, but they do not always occur (81) and are therefore not reliable for diagnosing FIV infection.

During the mating season preceding her positive test in February 2017, NAIA had a neck bite wound, was in suboptimal general condition, and showed a p24 band in the WB, suggesting that she may have been in an early infection stage at the time of her first field capture in 2016 (3). Since she was likely ADIN's mate, he may have infected her through the copulation neck bite but being infectious would have implied that he was viremic, which presupposes a recent infection and suggests intraspecific transmission among lynx within a small geographical radius within a few months in 2016. At the time of euthanasia, SENI and NAIA only showed a p24 band reaction, like it often occurs in domestic cats in the late phase of infection and can be due either to immunosuppression or to sequestration of antibodies by virus-antibody complex (3). These observations suggests a rapid disease course of less than a year for the three lynx, something that has been observed in domestic cats infected with a particular virulent FIV strain of subtype C, though in this case rapidly progressive disease was associated with high viral load (83). However, the latter was not the case in the three lynxes as we were unable to detect FIV by qPCR at any time. This raises the question of the specificity of the used molecular detection methods and suggests the implication of a serological cross-reacting virus that was not detected by the FIV PCR protocols used.

Whether FIV or an antigenically related virus, given that SENI had only recently arrived in proximity of ADIN's territory when he was caught in 2017, he likely became infected after ADIN was removed from the field. Therefore, we assumed that if all three cases were really linked, another individual in this area must have been infected, too, likely a resident male defending his territory, such as CARV or WENG (Figure 3), which were both FIV-negative in 2013 and 2016. respectively. However, this hypothesis was refuted by subsequent follow up of these two males. CARV was recaptured in February 2019 and was still FIV-negative and apparently healthy. WENG could not be re-captured for testing but monitoring by radiotelemetry suggested he remained healthy until January 2019, when his occurrence was confirmed for the last time. Nevertheless, other nonidentified lynx may have crossed ADIN's way at that time and served as a link between him and SENI. Since lynx have a solitary lifestyle and intraspecific interactions outside the family group occur mainly during the mating season (45), if the disease course was as fast as it seemed to have been for the three observed lynx, this potential fourth case may have died before infecting other conspecifics.

Our data suggest that FIV or a serological cross-reacting virus had emerged in the Jura lynx population, raising the question of the origin of the virus. Strains of FIV are largely species specific but cross-species transmission can occur (51) and spillover from domestic cats to wild felids have been documented in guignas (kodkod, *Leopardus guigna*) (10) and a Tsushima cat (*Felis bengalensis euptilura*) (11), and there was no indication of previous FIV exposure in lynx. Therefore, the most likely explanation would be a spillover from another host such as a domestic cat. Although uncommon, lynx sometimes fight with, kill, and may eventually eat domestic cats, as previously documented in the Jura Mountains (84), providing opportunities for pathogen transmission. As a matter of fact, field data suggested that SENI had killed two domestic cats between his first and second capture. There are no current data on FIV prevalence in domestic cats in Switzerland but a study published in 1990 found the FIV prevalence in domestic cats to be <1% in apparently healthy and <4% in sick animals (85), and a study with a sample size of >17,000 cats performed in Germany (which is adjacent to Jura North, Figure 10) from 1993 to 2002 also found a low prevalence of 3.2%, which had remained stable over the study period (86). There are no current FIV survey data from domestic cats in Switzerland, but prevalence is assumed to be still low except for certain hotspot areas with FIV infections in free-roaming domestic cats (unpublished author data). Overall, these data on domestic cats and cat-lynx interactions suggests a very low probability of lynx infection by FIV or another similar virus from a domestic cat. Although separate transmission events cannot be excluded, this low risk and especially the spatiotemporal proximity of the three cases presented here is striking and suggests a common infection source.

The WB results in these three lynx revealed that former investigations by ELISA were not reliable and that the applied POC test is likely useless for lynx. In agreement with our observations, the POC test delivered negative results for guignas that tested FIV-positive by PCR (10). This questions the result validity of former studies on wild felids relying only on these diagnostic methods. We therefore recommend to systematically use the WB to test felids for FIV antibodies.

Because the FIV matrix p24 may cross-react with other antibodies (16), we consider that detection of antibodies also to the FIV capsid protein p15 is required to declare an animal seropositive to FIV, even if in the acute phase, and again in the terminal phase, only p24 may react in the test (Figure 1). Our study revealed that many free-ranging lynx show a reaction to p24. They were apparently healthy animals scattered across the three populations and found in all study years, sex, and age groups. Additionally, part of the lynx sampled multiple times changed status over time. Although a reaction related to an early infection in part of the lynx cannot be excluded, these observations point at an unspecific reaction.

None of the lynx tested for FIV for the first time were found FIV-seropositive (0% prevalence) but three individuals from the Jura population had seroconverted by the time of their second capture. Given the estimated population size in the Swiss Jura during our study period (60 +/−7 individuals in 2017; latest available numbers from www.kora.ch), a sample size of 47 negative lynx out of 60 gives a maximal possible prevalence of 5% for this population, or maximum three positive individuals, with a confidence level of 95%, and accepted error of 0.5% and assuming a perfect test (www.winepi.net). Although sampling had occurred over a long time period, it shows that our sample size was good especially when considering that exactly three individuals were found seropositive later on. However, the largest part of the Jura lynx population is in neighboring France, meaning that the whole population is larger and therefore the level of uncertainty actually higher than suggested by our estimation. We may have increased our sample size by using archived samples of dead lynx. However, since former investigation of the specificity of serological tests in domestic cats when using hemolytic serum samples, like is typically collected from carcasses, have shown that prevalences may be under- or overestimated by 7–10% [Leutenegger et al. (17)], we preferred to work with a smaller sample size than taking the risk of obtaining false positive results. Last but not least, while SENI and ADIN had been clearly negative at earlier time points, at her first examination NAIA showed a p24 band. We found numerous lynx with a p24 band, including many with a changing status in captivity. These observations are in agreement with unspecific reactions (3, 16) and may have been also the case of NAIA. In view of her second WB results, we cannot exclude that part of the lynx with a p24 band may have become positive in the WB later on, which adds uncertainty regarding the potential occurrence of more lynx infected with FIV in the population. Assuming that viral transmission among lynx occurs mainly through mating bites and considering that maternal antibodies can be found in cats younger than 6 months old (14), it is reassuring that none of the tested newborn kittens were found positive, even if the sample size of this age group was very limited.

Despite persistent efforts to detect replicating virus or the genome of FIV in lynx samples, all attempts failed. Molecular diagnostics (PCR) are known to have poor specificity and sensitivity for FIV, the first due to a high genetic diversity of FIV, the second due to possible different performances of the qPCR and conventional PCR tests, in conjunction with very low viral loads (3, 5). Low viral loads may also have hampered NGS attempts, which were successful using samples from an FIV-infected domestic cat (data not shown). Although MLR should have reactivated latent virus, none could be detected from MLR supernatants neither by RT-qPCR nor by NGS, as it was the case for the positive control cat. The antibodies detected by WB using FIV (5) may have been due to reaction to FIV originating from a domestic cat (see above, cross-species transmission) or due to a cross-reaction with a retrovirus other than FIV, which could not be detected by the applied FIV PCR protocols. Cross-reactive antibodies to FIV are widespread in free-ranging populations of wild felids (6–9), and lentivirus detection was successful in some of them (9).

There was a very limited RT activity in the lynx samples when compared to the result of the latent infected domestic cat. Low activity could also be ascribed to an unrevealed endogenous retrovirus (ERV) (87, 88). Furthermore, emergence of three FIV-seropositive lynx by WB in the same area (Jura North) nearly at the same time (2016–2017) can hardly be coincidental. Last but not least, these lynx clearly developed unusual health problems although the disease picture was not clearly attributable to FIV. Therefore, either the reactivation of the virus by MLR failed, for reasons yet unidentified by the authors, or this RT activity was the result of an unspecific cross-reaction. Further efforts may be done by NGS approach using the processed samples that were stored for further analysis, such as bone marrow and lymph node cell cultures.

Overall, extreme stress during quarantine may explain many, but likely not all, health issues in these lynx. With or without infection as a predisposing factor, the findings in the three FIV-seropositive lynx corroborate our former recommendations to keep quarantine duration to a minimum for wild-born lynx, as an important measure to preserve health and animal welfare (23).

In conclusion, although we were not able to confirm FIV infection by virus isolation and molecular characterization, our observations suggest emergence of a lentivirus with antigenic and pathogenic similarities to FIV in the lynx population of Jura North. Post-mortem analysis revealed that euthanasia was appropriate in the three affected lynx despite unconclusive diagnosis. If such a situation would occur again in the future, euthanasia would be recommended for lynx with signs of health impairment or and/or with documented viremia. Adequate captive conditions required for prolonged observation of adult wild-born lynx are unlikely to be found. Thus, in absence of both disease signs and viremia, euthanasia would be ethically questionable and these lynx may be repatriated fitted with a radio-collar, assuming that the initial FIV infection phase would be over and infection risk low. Health risk in wildlife translocation is never zero, and decisions are balancing acts between disease risks and conservation interests. Our experiences underline the necessity to continue including FIV in pathogen screenings of free-ranging European wild felids, the importance of lynx health monitoring, and the usefulness of health protocols in wildlife translocations.

## Data Availability Statement

The original contributions presented in the study are included in the article/Supplementary Material, further inquiries can be directed to the corresponding author.

## Ethics Statement

Ethical review and approval was not required for the animal study because it was not required for this specific work on FIV. Capture and sampling of wild animals were done with the necessary authorizations of the Federal Office of the Environment (capture of a species protected by federal law; permits issued on 17.01.2001, 314.12.2001, 05.09.2002, 26.01.2004, 24.01.2007, 01.03.2011, 17.12.2013, 30.08.2017, and 22.07.2020) and of the commission for animal experimentation required in Switzerland to immobilize and sample protected wildlife for research purposes (permits Nr 66/97, 8/00, 89/02 360.431.00, SO57052, BE12/07, 109/10, 111/13+, 3/17, 3/17+, BE61/20).

## Author Contributions

MPRD carried out the risk analysis for, and coordinated the veterinary supervision of, the translocation projects incl. disease management strategy development. MLM and RHL advised risk analysis, protocol development, and disease management, supervised most laboratory analyses, and provided expertise on FIV infection. JK and CB developed the protocols, performed the sequencing, and analyzed NGS data. JBH and JB developed the protocols and performed the PERT assays. MPRD, IM, SRRP, MP, and CBW contributed to lynx captures. MPRD, IM, SRRP, and MP carried out clinical examinations and sampling. MW was responsible for animal care at the Swiss quarantine station. CBW organized the first investigations on FIV prior to the current lynx health surveillance program and provided data on radio-collared lynx and expertise on lynx biology. SRRP, IM, FO, MPRD, AKH, FK, and AP carried out or contributed to pathological examinations. ME-S carried out in-depth dental examination in ADIN. BR supervised the hematology and blood chemistry analyses and provided expertise in clinical pathology. MPRD, IM, and MLM analyzed the data. IM and MPRD prepared the tables. SRRP, MPRD, and CBW produced the figures. MPRD and MLM drafted the manuscript. All authors critically read and approved the final version of the manuscript.

## Funding

Translocation projects were funded by the Swiss Federal office of Environment (FOEN), which also support health monitoring of Swiss lynx by the Center for Fish and Wildlife Health and population monitoring by the KORA. Sampling and serological analyzes for lynx captured between April 2020 and April 2021 were possible thanks to a grant of the Foundation Haldimann. Virological analyses were possible thanks to additional financial contributions of the Foundation Galli-Valerio, the FOEN, the Clinical Laboratory, the Institute of Virology, Vetsuisse Faculty Zurich, and the Institute of medical Virology of the University of Zurich.

## Conflict of Interest

The authors declare that the research was conducted in the absence of any commercial or financial relationships that could be construed as a potential conflict of interest.

## Publisher's Note

All claims expressed in this article are solely those of the authors and do not necessarily represent those of their affiliated organizations, or those of the publisher, the editors and the reviewers. Any product that may be evaluated in this article, or claim that may be made by its manufacturer, is not guaranteed or endorsed by the publisher.
