# Supplementary material for: Management of Suspected Cases of Feline Immunodeficiency Virus Infection in Eurasian Lynx (Lynx lynx) During an International Translocation Program
Source: Front Vet Sci. 2021 Oct 25;8:730874. doi: 10.3389/fvets.2021.730874 (PMC8573149; doi:10.3389/fvets.2021.730874)
Supplement: Supplementary file 1 [file Data_Sheet_1.docx]

Supplementary Material

# Knowledge on feline immunodeficiency virus relevant to disease management in lynx

## 1.1 Virus characteristics, occurrence, and hosts

# Feline immunodeficiency virus (FIV) is a retrovirus of the genus *Lentivirus*, which is closely related to human immunodeficiency virus causing the Acquired Immunodeficiency Syndrome (AIDS) in humans and characterised by similar disease mechanisms (2–5). Five FIV subtypes have been described worldwide (A to E), though new subtypes F and U-NZenv have been recently proposed. Most FIV identified so far belong to subtypes A or B, both of them occur in Europe (4,6). FIV subtypes are not equally common everywhere, and geographic clustering is evident. Moreover, co-infection with two different strains may occur (5). Furthermore, FIV mutates rapidly and shows a high overall genetic diversity. These factors make virus detection by molecular techniques (PCR) challenging (4,6).

FIV occurs in domestic cat populations worldwide. The frequency of infection was estimated to vary from 1-16% in healthy cats to 44% in sick cats (4), but current data is missing for many geographic regions. Cross-reactive antibodies to FIV occur in a number of free-ranging wild felids (2,3,7–9). FIV strains have also been detected in numerous wild felids, for example the puma (*Puma concolor*), lion (*Panthera leo*), leopard (*Panthera pardus*) and Pallas’ cat (*Otocolobus manul*) (1,7,8,10) but the clinical significance of these infections is controversial. There are several FIV strains that are largely species-specific, although this does not exclude occasional cross-species virus transmission (9,11). Cross-species transmission events can be singular, dead-end events or can result in viral replication and spread in the new species. Strains from wild felids may have a lower disease-causing potential since the virus and host may be adapted to each other in such a way that the infection has no or minimal negative effects on the host (2,7, 12,13). Meanwhile, it has been proposed to talk about FIVs as complex retroviruses with a propensity for rapid mutation and recombination leading to a high rate of viral evolution (1,12).

**1.2 Infection dynamics and disease phases**

FIV infection is characterized by the integration of a DNA copy of the viral RNA (called provirus), which results in life-long infection typically including three main phases (14). The first, acute phase immediately follows infection. Although great variations in virus and antibody dynamics have been documented (15), more recent knowledge shows that under experimental conditions, viremia becomes detectable within 2 weeks post infection, with a peak after 8-12 weeks (4). In parallel (2-4 weeks after infection, sometimes later), antibodies against FIV appear. Viral invasion of brain tissues also start during the acute phase, as early as within the first week following infection by intra-venous challenge (16). Clinical signs (anorexia, lethargy, fever) may develop during this initial phase but are usually mild and disappear rapidly. A change in blood count (neutropenia) and generalized swelling of the peripheral lymph nodes can also be observed. With the reduction of viruses in blood, infected cats become clinically healthy again, although generalized lymphadenopathy may last for weeks up to months (4,17). During this second and longest, asymptomatic or subclinical phase, viral replication is very limited (14). The dormant (latent) form of the virus, the provirus, persists integrated in the host’s DNA and cannot be reached by the immune system. Infected animals can remain healthy for years. Depending on the virus strain it is also possible that the animals never become ill. Nevertheless, typically the body's defense cells (CD4+ T lymphocytes) decrease slowly (typically with an inversion of the CD4+/CD8+ T cell ratio), starting already a few weeks after infection, and this progressive deterioration of T-cell function leads to an increase of viral replication, lymphocytopenia, lymph node atrophy and immunodeficiency. This third and last phase begins with the so-called AIDS-related complex, which may last several months to years and is associated with chronic secondary infections by non-opportunistic pathogens, and finally develops into the terminal AIDS-like phase (Feline acquired immunodeficiency syndrome, FAIDS), which can also last for months and is characterized by a range of disorders, including severe secondary infections caused by opportunistic pathogens. The disease typically ends with a euthanasia (18–20) unless natural death occurs. These disease stages and characteristics have been mainly described under experimental conditions in domestic cats and it is unclear to what extent they are also found under field conditions. Variations probably exist depending on the virus isolates.

**1.3 Disease signs and lesions**

Most disease signs in FIV infected cats are the result of secondary infections or neoplasia due to FIV induced immunodeficiency or of immune stimulation (immune mediated diseases). Typical findings include fever, lethargy, signs of enteritis, stomatitis, dermatitis, conjunctivitis, respiratory tract pathologies, generalized lymphadenopathy (usually atrophy or a mixed pattern), weight loss, and renal disease together with an increased protein content in blood and urine (proteinemia and proteinuria). Leukopenia, anemia or both may be present, especially during the late stages of disease. Furthermore, bacterial, viral, parasitic, and fungal infections (e.g., unusual, or particularly severe parasitic skin diseases such as demodicosis, pediculosis) can occur as well as tumors (4,15,21,22). Neurological disorders, mainly of behavioral nature, have also been reported. Reproductive failure may occur in infected cats when the virus has reached the placenta and fetuses (4,21–23). Immediately after initial infection, FIV quickly disseminates to many anatomical compartments within the host including lymphoid organs, gastrointestinal tract, and brain, whereas brain tropism strongly varies with the FIV strain. Less commonly, FIV has also been identified in lung, liver, kidney and/or salivary glands, as well as the vaginal mucosa (24). At histopathological examination, neuropathies (25), nephropathies (26,27), and alterations of lymphoid organs (7) are typically seen, together with lesions related to secondary infections. Like for AIDS in humans, the broad range of disease signs and post-mortem pathological changes is a result of health impairments secondary to a progressive disruption of the host’s immune functions, which makes it particularly difficult to achieve an etiologic diagnosis. This is further complicated by the fact that internal (e.g., sex and age at the time of first exposure) and external factors (such as environmental stress) can influence the infection course and disease progression (28).

The occurrence of disease signs or lesions doubtlessly associated with FIV infection has not been documented in wildlife, however, this challenge is also encountered with domestic cats (29). FIV infections have been studied in particular in the African lion, the puma, the bobcat, in which documented high seroprevalences and absence of associated health problems are indeed believed to indicate co-evolution of virus and host (1,3,8,13,30,31). Antibodies to FIV and FIV sequences were also found in guignas (*Leopardus guigna*) from Chile and a Tsushima cat (*Felis bengalensis euptilura*) from Japan in absence of associated disease (9,11). However, a study comparing FIV-positive and FIV-negative lions demonstrated that FIV-associated clinical and histological alterations known to occur in domestic cats, humans and macaques infected with their respective immunodeficiency viruses, and consistent with immune deficiencies, are more likely to be found in FIV-positive lions (7). To convincingly demonstrate an association between FIV infection and disease signs is particularly challenging because the typical feature of virus-induced immunodeficiencies is the lack of specific signs and lesions (29), with disease and death due to secondary infections, if ever occurring. An assessment is even more difficult in wild felids because experimental trials are not an option in protected species (32).

**1.4. Diagnostics**

The typical infection dynamics of FIV causes important challenges for diagnostics. Firstly, initial viremia is short and occurs before antibodies are detectable. Secondly, viremia drops below detection level once the antibody response has developed. Lastly, while both viremia and antibodies should be detectable and clinical signs can be expected in the terminal phase, virus reactivation and associated AIDS-like condition do not necessarily occur. In addition, not all FIV subtypes or virus isolates may be detected by specific PCR, and antibodies to some virus subtypes or strains may not be detected by ELISA (4,6). Serology is actually the method of choice for the diagnosis of a FIV infection because of the strong and consistent host antibody response detectable during the entire infection course, but reliable testing requires a Western blot (WB), which is considered the gold standard (6,14). A WB result is judged positive and reliable if two bands with a molecular weight of 15 kDa (capsid protein p15) and 24 kDa (matrix protein p24) are recognizable on the blotting strip (33). Since FIV p24 may cross-react with other antibodies (34), detection of antibodies also to p15 is required to declare an animal seropositive to FIV. However, in the early acute phase, and again in the terminal phase, only p24 may react in the test (Figure 10), suggesting a lack of anti-FIV antibodies early after infection (before seroconversion) or in the terminal disease stage due to immunodeficiency. Alternatively, antibodies may be undetectable in case of high virus load due to sequestration of antibodies in immune complex formation with the virus (4). Therefore, it is recommended to re-test domestic cats showing a p24 band two to three months later (6).

A PCR negative result in a seropositive animal may be explained by the presence of a FIV subtype not recognised by the PCR, rather than by the absence of FIV infection (4). Very low proviral loads would likely also lead to a negative PCR result.

**1.5. Transmission**

Feline immunodeficiency virus is mainly excreted in saliva and transmission occurs mostly through bites (territorial fights or mating bites). Transmission from the dam to the kittens during pregnancy and lactation may occur, mostly during the acute phase of FIV infection in the dam. Other routes of infection have hardly been documented under natural conditions (4,15,35–38). Thus, a fresh or healing bite wound can be a sign of a recent FIV exposure and may be present in animals before antibodies and viruses can be detected. Transmission most likely occurs during viremia and is thus expected to be limited to the initial and last phases of infection.

**1.6. Prevention**

The virus survives only minutes outside the host and is sensitive to all disinfectants including normal soap. In domestic cats, positive animals shall be confined indoors to prevent the infection of other cats (39). There is no vaccine against FIV available in Europe (4).

**References**

1. Lee JS, Bevins SN, Serieys LEK, Vickers W, Logan KA, Aldredge M, Boydston EE, Lyren LM, McBride R, Roelke-Parker M, et al. Evolution of puma lentivirus in bobcats (*Lynx rufus*) and mountain lions (*Puma concolor*) in North America. *J Virol* (2014) **88**:7727–7737. doi:10.1128/JVI.00473-14

2. Brown EW, Miththapala S, O’Brien SJ. Prevalence of Exposure to Feline Immunodeficiency Virus in Exotic Felid Species. *Journal of Zoo and Wildlife Medicine* (1993) **24**:357–364.

3. Troyer JL, Pecon-Slattery J, Roelke ME, Johnson W, VandeWoude S, Vazquez-Salat N, Brown M, Frank L, Woodroffe R, Winterbach C, et al. Seroprevalence and genomic divergence of circulating strains of feline immunodeficiency virus among Felidae and Hyaenidae species. *J Virol* (2005) **79**:8282–8294. doi:10.1128/JVI.79.13.8282-8294.2005

4. Hosie MJ, Addie D, Belák S, Boucraut-Baralon C, Egberink H, Frymus T, Gruffydd-Jones T, Hartmann K, Lloret A, Lutz H, et al. Feline immunodeficiency. ABCD guidelines on prevention and management. *J Feline Med Surg* (2009) **11**:575–584. doi:10.1016/j.jfms.2009.05.006

5. Reggeti F, Bienzle D. Feline immunodeficiency virus subtypes A, B and C and intersubtype recombinants in Ontario, Canada. *J Gen Virol* (2004) **85**:1843–1852. doi:10.1099/vir.0.19743-0

6. Frankenfeld J, Meili T, Meli ML, Riond B, Helfer-Hungerbuehler AK, Bönzli E, Pineroli B, Hofmann-Lehmann R. Decreased sensitivity of the serological detection of Feline Immunodeficiency Virus infection potentially due to imported genetic variants. *Viruses* (2019) **11**: doi:10.3390/v11080697

7. Roelke ME, Brown MA, Troyer JL, Winterbach H, Winterbach C, Hemson G, Smith D, Johnson RC, Pecon-Slattery J, Roca AL, et al. Pathological manifestations of feline immunodeficiency virus (FIV) infection in wild African lions. *Virology* (2009) **390**:1–12. doi:10.1016/j.virol.2009.04.011

8. Brown MA, Munkhtsog B, Troyer JL, Ross S, Sellers R, Fine AE, Swanson WF, Roelke ME, O’Brien1 SJ. Feline immunodeficiency virus (FIV) in wild Pallas’ cats. *Vet Immunol Immunopathol* (2010) **134**:90. doi:10.1016/j.vetimm.2009.10.014

9. Sacristán I, Acuña F, Aguilar E, García S, José López M, Cabello J, Hidalgo‐Hermoso E, Sanderson J, Terio KA, Barrs V, et al. Cross‐species transmission of retroviruses among domestic and wild felids in human‐occupied landscapes in Chile. *Evol Appl* (2021) **14**:1070–1082. doi:10.1111/eva.13181

10. Olmsted RA, Langley R, Roelke ME, Goeken RM, Adger-Johnson D, Goff JP, Albert JP, Packer C, Laurenson MK, Caro TM. Worldwide prevalence of lentivirus infection in wild feline species: epidemiologic and phylogenetic aspects. *J Virol* (1992) **66**:6008–6018. doi:10.1128/JVI.66.10.6008-6018.1992

11. Nishimura Y, Goto Y, Yoneda K, Endo Y, Mizuno T, Hamachi M, Maruyama H, Kinoshita H, Koga S, Komori M, et al. Interspecies transmission of Feline Immunodeficiency Virus from the domestic cat to the Tsushima cat (Felis bengalensis euptilura) in the Wild. *J Virol* (1999) **73**:7916–7921.

12. Lee J, Malmberg JL, Wood BA, Hladky S, Troyer R, Roelke M, Cunningham M, McBride R, Vickers W, Boyce W, et al. Feline Immunodeficiency Virus cross-species transmission: implications for emergence of new lentiviral infections. *J Virol* (2017) **91**: doi:10.1128/JVI.02134-16

13. Troyer JL, Roelke ME, Jespersen JM, Baggett N, Buckley-Beason V, MacNulty D, Craft M, Packer C, Pecon-Slattery J, O’Brien SJ. FIV diversity: FIVPle subtype composition may influence disease outcome in African lions. *Vet Immunol Immunopathol* (2011) **143**:338–346. doi:10.1016/j.vetimm.2011.06.013

14. Westman ME, Malik R, Norris JM. Diagnosing feline immunodeficiency virus (FIV) and feline leukaemia virus (FeLV) infection: an update for clinicians. *Aust Vet J* (2019) **97**:47–55. doi:10.1111/avj.12781

15. Hartmann K. Feline immunodeficiency virus infection: an overview. *Vet J* (1998) **155**:123–137. doi:10.1016/S1090-0233(98)80008-7

16. Ryan G, Klein D, Knapp E, Hosie MJ, Grimes T, Mabruk MJEMF, Jarrett O, Callanan JJ. Dynamics of viral and Proviral Loads of Feline Immunodeficiency Virus within the feline central nervous system during the acute phase following intravenous infection. *J Virol* (2003) **77**:7477–7485. doi:10.1128/JVI.77.13.7477-7485.2003

17. Torten M, Franchini M, Barlough JE, George JW, Mozes E, Lutz H, Pedersen NC. Progressive immune dysfunction in cats experimentally infected with feline immunodeficiency virus. *J Virol* (1991) **65**:2225–2230.

18. Reperant LA, Osterhaus ADME. “Retrovirus infections,” in *Infectious diseases of wild mammals and birds in Europe,* Gavier-Widén D, Duff JP, Meredith A (eds) (Oxford, United Kingdom: Wiley-Blackwell), 219–224.

19. Lutz H. “Virusinfektionen,” in *Krankheiten der Katze,* Horzinek MC, Schmidt V, Lutz H (eds) (Stuttgart, Germany: Enke Verlag), 107–155.

20. Hopper CD, Sparkes AH, Harbour DH. “Feline immunodeficiency virus,” in *Feline medicine and therapeuthics,* Chandler EA, Gaskel CJ, Gaskell RM (eds) (Oxford, United Kingdom: Blackwell Science), 488–505.

21. Hartmann K. Clinical aspects of feline retroviruses: a review. *Viruses* (2012) **4**:2684–2710. doi:10.3390/v4112684

22. Meeker RB, Hudson L. Feline Immunodeficiency Virus neuropathogenesis: A model for HIV-induced CNS inflammation and neurodegeneration. *Vet Sci* (2017) **4**: doi:10.3390/vetsci4010014

23. Hartmann K. Clinical aspects of feline immunodeficiency and feline leukemia virus infection. *Vet Immunol Immunopathol* (2011) **143**:190–201. doi:10.1016/j.vetimm.2011.06.003

24. Eckstrand CD, Sparger EE, Murphy BG. Central and peripheral reservoirs of feline immunodeficiency virus in cats: a review. *Journal of General Virology* (2017) **98**:1985–1996. doi:10.1099/jgv.0.000866

25. Ryan G, Grimes T, Brankin B, Mabruk MJEMF, Hosie MJ, Jarrett O, Callanan JJ. Neuropathology associated with feline immunodeficiency virus infection highlights prominent lymphocyte trafficking through both the blood-brain and blood-choroid plexus barriers. *J Neurovirol* (2005) **11**:337–345. doi:10.1080/13550280500186445

26. Poli A, Tozon N, Guidi G, Pistello M. Renal alterations in feline immunodeficiency virus (FIV)-infected cats: a natural model of lentivirus-induced renal disease changes. *Viruses* (2012) **4**:1372–1389. doi:10.3390/v4091372

27. Poli A, Abramo F, Taccini E, Guidi G, Barsotti P, Bendinelli M, Malvaldi G. Renal involvement in feline immunodeficiency virus infection: a clinicopathological study. *Nephron* (1993) **64**:282–288. doi:10.1159/000187327

28. Bęczkowski PM, Litster A, Lin TL, Mellor DJ, Willett BJ, Hosie MJ. Contrasting clinical outcomes in two cohorts of cats naturally infected with feline immunodeficiency virus (FIV). *Vet Microbiol* (2015) **176**:50–60. doi:10.1016/j.vetmic.2014.12.023

29. Bendinelli M, Pistello M, Lombardi S, Poli A, Garzelli C, Matteucci D, Ceccherini-Nelli L, Malvaldi G, Tozzini F. Feline immunodeficiency virus: an interesting model for AIDS studies and an important cat pathogen. *Clin Microbiol Rev* (1995) **8**:87–112.

30. Munson L, Terio KA, Ryser-Degiorgis M-P, Lane PE, Courchamp F. “Wild felid diseases: conservation implications and management strategies,” in *Biology and conservation of wild felids* (Oxford, New York, United States: Oxford University Press), 237–259.

31. Bevins SN, Carver S, Boydston EE, Lyren LM, Alldredge M, Logan KA, Riley SPD, Fisher RN, Vickers TW, Boyce W, et al. Three pathogens in sympatric populations of pumas, bobcats, and domestic cats: implications for infectious disease transmission. *PLoS One* (2012) **7**: doi:10.1371/journal.pone.0031403

32. White J, Stickney A, Norris JM. Feline immunodeficiency virus: disease association versus causation in domestic and nondomestic felids. *Vet Clin North Am Small Anim Pract* (2011) **41**:1197–1208. doi:10.1016/j.cvsm.2011.07.003

33. Lutz H, Arnold P, Hübscher U, Egberink H, Pedersen N, Horzinek MC. Specificity assessment of feline T-lymphotropic lentivirus serology. *Zentralbl Veterinarmed B* (1988) **35**:773–778. doi:10.1111/j.1439-0450.1988.tb00559.x

34. Egberink HF, Lutz H, Horzinek MC. Use of western blot and radioimmunoprecipitation for diagnosis of feline leukemia and feline immunodeficiency virus infections. *J Am Vet Med Assoc* (1991) **199**:1339–1342.

35. Matteucci D, Baldinotti F, Mazzetti P, Pistello M, Bandecchi P, Ghilarducci R, Poli A, Tozzini F, Bendinelli M. Detection of feline immunodeficiency virus in saliva and plasma by cultivation and polymerase chain reaction. *J Clin Microbiol* (1993) **31**:494–501.

36. O’Neil LL, Burkhard MJ, Hoover EA. Frequent perinatal transmission of feline immunodeficiency virus by chronically infected cats. *J Virol* (1996) **70**:2894–2901.

37. O’Neil LL, Burkhard MJ, Diehl LJ, Hoover EA. Vertical Transmission of Feline Immunodeficiency Virus. *AIDS Research and Human Retroviruses* (1995) **11**:171–182. doi:10.1089/aid.1995.11.171

38. Reynolds JJH, Carver S, Cunningham MW, Logan KA, Vickers W, Crooks KR, VandeWoude S, Craft ME. Feline immunodeficiency virus in puma: Estimation of force of infection reveals insights into transmission. *Ecol Evol* (2019) **9**:11010–11024. doi:10.1002/ece3.5584

39. Little S, Levy J, Hartmann K, Hofmann-Lehmann R, Hosie M, Olah G, Denis KS. 2020 AAFP Feline retrovirus testing and management guidelines. *Journal of Feline Medicine and Surgery* (2020) **22**:5–30. doi:10.1177/1098612X19895940
