# Supplementary material for: Management of Suspected Cases of Feline Immunodeficiency Virus Infection in Eurasian Lynx (Lynx lynx) During an International Translocation Program
Source: Front Vet Sci. 2021 Oct 25;8:730874. doi: 10.3389/fvets.2021.730874 (PMC8573149; doi:10.3389/fvets.2021.730874)
Supplement: Supplementary file 2 [file Data_Sheet_2.docx]

Supplementary Material

# Virological methods

**2.1 Nucleic acid extractions**

Total Nucleic Acids (TNA) were purified from EDTA-anticoagulated whole blood, swab samples and cell culture supernatants using the MagNA Pure Total Nucleic Acids Isolation Kit (Roche Diagnostics, Rotkreuz, Switzerland) as previously described (1).

**2.2 Conventional nested and semi-nested PCRs for the detection of FIV Provirus**

A semi-nested conventional PCR was performed that amplified a 470 bp-long sequence from the FIV subtype A and B *gag* gene using primers previously described (2,3) with some modifications. Briefly the final reaction volume was 25 µl in both rounds with 2.5 µl 10x Reaction Mix, 6.25 Units Taq Polymerase (Sigma-Aldrich), 1 µM of each primer (Z201and Z202 for the first round and Z201 and Z203 for the second round), 0.2 mM dNTPs (ThermoFisher Scientific, Basel, Switzerland) and 1.5 mM MgCl_2_. 1 μl of the first round PCR was used in the second round as template. Cycling conditions: five minutes at 95°C, followed by 35 cycles of 30 seconds at 95°C, 30 seconds at 58°C and 1 minute at 72°C and a final elongation step of 10 minutes at 72°C. The PCR products from the second round of PCR, with an expected length of 470 bp, were analysed by agarose gel electrophoresis (2%). All bands of the expected length were purified (QIAQuick Gel Extraction Kit, QIAGEN, Hilden, Germany) and submitted for sequencing (Microsynth AG, Balgach, Switzerland). Another semi-nested conventional PCR, targeting the 5’ LTR and following gag region of FIV A, B and C subtypes was performed (4) with some modifications. The final reaction volume was 25 µl in both reactions with 2.5 µl 10x Reaction Mix, 3.9 Units Taq Polymerase (Sigma-Aldrich), 1 µM of each primer (LTR1.122f and Gag1.1409r), 0.2 mM dNTPs (ThermoFisher scientific) and 1.5 mM MgCl_2_. Cycling conditions: five minutes at 95°C, followed by 40 cycles of 45 seconds at 95°C, 30 seconds at 60°C and 1.5 minute at 72°C and a final elongation step of 10 minutes at 72°C. 1 μl of the first round PCR was used in the second round as template with the same reaction composition and Primers LTR2.285f and Gag1.1409r and the same cycling condition with exception of the annealing temperature (64°C). The PCR products from the second round of PCR, with an expected length of 1127 bp, were analysed by agarose gel electrophoresis (2%). A further nested PCR targeting the Pol gene (ca 500 bp) was performed as previously described (5) with some modifications. Briefly, the final reaction volume was 20 µl in both reactions with 4 µl 5x Phusion ™ HF buffer (Finnzymes, Espoo, Finland), 0.4 Units Phusion™ Hifi DNA Polymerase (Finnzymes), 0.5 µM of each primer (FIV-1258F and FIV-1260R) and 0.2 mM dNTPs (ThermoFisher scientific). Cycling conditions: three minutes at 98°C, followed by 40 cycles of 15 seconds at 98°C, 30 seconds at 45°C and 45 seconds at 72°C and a final elongation step of 5 minutes at 72°C. 2 μl of the first round PCR was used in the second round as template with the same reaction composition and Primers FIV-1259F and FIV-1261R and the same cycling condition with exception of the annealing temperature (50°C). The PCR products from the second round of PCR, with an expected length of 1127 bp, were analysed by agarose gel electrophoresis (2%).

**2.3 Virus characterization/isolation**

Peripheral Blood Mononuclear Cells (PBMC) were isolated from 5 ml heparin-anticoagulated whole blood by Ficoll (Histopaque-1077, Sigma-Aldrich, Switzerland) density gradient centrifugation as previously described (6) and grown in RPMI complete medium (RPMI 1640 (Sigma-Aldrich) supplemented with 10% fetal calf serum (FCS, Gibco), 1% Antibiotics/Antimycotic (Gibco 15240-062, 10’000 units/ ml Penicillin G sodium, 10’000 ug/ml Streptomycin sulphate, 25 ug/ml Amphotericin B), 1% Glutamine (200 mM L-Glutamine (Gibco 25030-024)) and stimulated with 10 mg/ml Concanavalin A (ConA, Sigma-Aldrich) for 18-24h at 37°C with 5% CO_2_. After 24h fresh medium and 100 units/ml of recombinant human interleukin-2 (IL-2, Sigma-Aldrich) were added to the cells and grown for more 24 h. Cells were collected by centrifugation resuspended in Recovery Cell Culture Freezing Medium (Gibco) and stored in liquid nitrogen until further use.

Coculture by mixing PBMC from FIV seropositive lynx and uninfected (WB-negative) lynx and/or specific pathogen-free (SPF) cats (blood donor cats, approved by the veterinary office of the Swiss Canton of Zurich ZH 226/16) were initiated to stimulate the reactivation of latent FIV by Mixed Lymphocyte Reaction (MLR), an *in vitro* assay in which leukocytes, from two genetically distinct individuals of the same or similar species, are cocultured resulting in cell blast transformation, DNA synthesis and proliferation (7,8). Briefly PBMC from uninfected animals (lynx and SPF cats) were thaw and grown at 37°C and 5% CO_2_ in RPMI complete medium (see above) containing 1% PHA for 72 hours. The medium was then replaced with IL-2 cell culture Medium (RPMI 1640 medium, 0.2 mmol/L Glutamine; 10% FCS, 1 ng/mL IL-2; 5 pg/mL polybrene; 50 μmol/mL Mercaptoethanol; 0.8 μg/L Insulin; 5 μg/L Pyruvate; 1.32 μg/L Oxalacetate) and aliquoted into 2-3 wells of a 24-well plate (Greiner-Bio One, St. Gallen, Switzerland) at a concentration of at least 10^6^ cells/ml and grown during 20 days by replacing the IL-2 Medium each 3-4 day. Cells were then pooled and counted with a Neubauer cell counting chamber. The PBMC of the seropositive animals, three lynx and a latently FIV infected cat as positive control (from a previous experimental infection experiment (9), approved by the veterinary office of the Swiss Canton of Zurich (ZH197/89, ZH43/ 90, ZH66/91, ZH131/91, ZH329/91, ZH56/95), were thawed and resuspended in IL-2 Medium. Cells were counted with a Neubauer cell counting chamber. A coculture of 1:5 vol/vol of infected to uninfected PBMC was set-up per well in a 24-well or 12-well plate depending on cell numbers (at least 3x10^5^ cells per well in a 24-well plate or 6x10^5^ cells per well in a 12-well plate). As negative controls PBMC of each animal alone and co-cultures of two uninfected animals were used. As positive controls co-cultures of a latently FIV infected cat with uninfected animals (lynx and cats) were used. Co-cultures were grown at 37°C and 5% CO_2_ for 6 weeks. Cell culture supernatant was collected weekly for PERT assay, NGS and RT-qPCR and replaced by fresh IL-2 Medium. Cell culture supernatants were stored by -80°C.

**2.3 Product-enhanced reverse transcriptase assay (PERT)**

Reverse transcriptase (RT) activity was assessed in supernatants of the co cultures at the Swiss National Center for Retroviruses, University of Zurich, Switzerland, using the PERT assay.

In this ultrasensitive method for the detection of reverse transcriptase (RT), the first step is to filter the culture supernatant sterilely to remove cell debris. Any virus particles present are sedimented and the pellet washed to remove inhibitors. Reverse transcriptase from the virus particles is released by detergents and detected in real-time RT-PCR. The PCR product formed is proportional to the input of RT molecules and almost reaches the sensitivity of a specific RT-PCR and is independent of the sequence of the virus genome under investigation (10, 11).

**2.4 Next Generation Sequencing (NGS)**

Supernatant of the coculture was further investigated using a metagenomic NGS approach (12). First, 300µl of the supernatant was filtrated through a 0.45µm syringe filter (Puradisc, Whatman GE Healthcare) and treated with RNase A (Sigma) and Micrococcal Nuclease (New England Biolabs). Subsequently, RNA and DNA were extracted using the QIAmp Viral RNA mini kit without carrier RNA (Qiagen). Then, the RNA was transcribed, the second strand was synthesized and amplified using sequence independent single primer amplification (SISPA). Libraries for sequencing were prepared using the NEBNext Ultra II DNA library prep kit and the NEBNext® Multiplex Oligos for Illumina® (both New England Biolabs) and sequenced on the Illumina NextSeq 500 system in a high output, paired end 2 x 150 nucleotide (nt) run at the Functional Genomics Center Zurich (FGCZ, Zurich, Switzerland). The generated raw reads were quality controlled and assembled to the inhouse database based on the RefSeq sequence collection and all additional complete FIV genomes available on NCBI Genbank at the time (www.ncbi.nlm.nih.gov) using the SeqMan NGen software ver 16. (Lasergene, DNAStar, USA).

**2.5. Processing of other samples**

Lymph nodes and spleen and collected at euthanasia were disrupted first with a sterile scalpel and then the suspension forced through 70 mm-pore cell strainer (Falcon, Sigma-Aldrich) to separate cells. The cell suspension was washed twice with Hank’s balanced Salt Solution (1x HBSS, Sigma-Aldrich) by centrifugation and finally resuspended in RPMI complete medium as described above and grown 24h at 37°C with 5% CO_2_. 200 ml of the cell suspension was collected for TNA isolation as described above. Cells were counted in a Sysmex cell counter and resuspended to a concentration between 1x10^6^ to 1x10^7^ cells/ml in Recovery Cell Culture Freezing Medium (ThermoFischer Scientific) and stored in liquid nitrogen until further use. For bone marrow samples to separate erythrocytes from white blood cells in a first attempt cells were overlayed on Ficoll (Histopaque-1077, Sigma-Aldrich) to be separated by density gradient centrifugation. As this was not working, erythrocytes were disrupted by resuspending the cell pellet in 1x RBC-lysis buffer (composition: 10x Buffer 1.5 M NH4Cl, 100mM NaHCO3, 10mM disodium EDTA, pH 7.4). The cell suspension was left 10 min at RT, mixed again and centrifuged at 1000 rpm for 10 min. Cells were resuspended in cell culture medium (RPMI 1640 + Mc Coy’s 5A 1:1 supplemented with 20% heat-inactivated FCS, 2 mM L-glutamine, 1x antibiotic-antimycotic and 0.25 µM Hydrocortisone) and incubated at 37°C and 5% CO_2_. 200 ml of the cell suspension was collected for TNA isolation as described above. Cells were resuspended in Recovery Cell Culture Freezing Medium (ThermoFischer Scientific) and stored in liquid nitrogen until further use.

**References**

1. Meli ML, Cattori V, Martínez F, López G, Vargas A, Simón MA, Zorrilla I, Muñoz A, Palomares F, López-Bao JV, et al. Feline Leukemia Virus and other pathogens as important threats to the survival of the critically endangered Iberian lynx (*Lynx pardinus*). *PLOS ONE* (2009) **4**:e4744. doi:10.1371/journal.pone.0004744

2. Allenspach K, Amacker M, Leutenegger CM, Hottiger M, Hofmann-Lehmann R, Hübscher U, Pistello M, Lutz H. [Quantification of proviral FIV DNA using competitive PCR]. *Schweiz Arch Tierheilkd* (1996) **138**:87–92.

3. Leutenegger CM, Hofmann-Lehmann R, Holznagel E, Cuisinier AM, Wolfensberger C, Duquesne V, Cronier J, Allenspach K, Aubert A, Ossent P, et al. Partial protection by vaccination with recombinant feline immunodeficiency virus surface glycoproteins. *AIDS Res Hum Retroviruses* (1998) **14**:275–283. doi:10.1089/aid.1998.14.275

4. Reggeti F, Bienzle D. Feline immunodeficiency virus subtypes A, B and C and intersubtype recombinants in Ontario, Canada. *J Gen Virol* (2004) **85**:1843–1852. doi:10.1099/vir.0.19743-0

5. Brown EW, Yuhki N, Packer C, O’Brien SJ. A lion lentivirus related to feline immunodeficiency virus: epidemiologic and phylogenetic aspects. *J Virol* (1994) **68**:5953–5968. doi:10.1128/JVI.68.9.5953-5968.1994

6. Kipar A, Leutenegger CM, Hetzel U, Akens MK, Mislin CN, Reinacher M, Lutz H. Cytokine mRNA levels in isolated feline monocytes. *Vet Immunol Immunopathol* (2001) **78**:305–315. doi:10.1016/s0165-2427(01)00240-9

7. Bain B, Lowenstein L, Kasakura S, Vas M. The mixedd leukocyte reaction and its potential use in tissue matching. *Bibl Haematol* (1968) **29**:617–627. doi:10.1159/000384673

8. Olmsted RA, Langley R, Roelke ME, Goeken RM, Adger-Johnson D, Goff JP, Albert JP, Packer C, Laurenson MK, Caro TM. Worldwide prevalence of lentivirus infection in wild feline species: epidemiologic and phylogenetic aspects. *J Virol* (1992) **66**:6008–6018. doi:10.1128/JVI.66.10.6008-6018.1992

9. Lehmann R, Franchini M, Aubert A, Wolfensberger C, Cronier J, Lutz H. Vaccination of cats experimentally infected with feline immunodeficiency virus, using a recombinant feline leukemia virus vaccine. *J Am Vet Med Assoc* (1991) **199**:1446–1452.

10. Leutenegger CM, Klein D, Hofmann-Lehmann R, Mislin C, Hummel U, Böni J, Boretti F, Guenzburg WH, Lutz H. Rapid feline immunodeficiency virus provirus quantitation by polymerase chain reaction using the TaqMan fluorogenic real-time detection system. *J Virol Methods* (1999) **78**:105–116. doi:10.1016/s0166-0934(98)00166-9

11. Klein D, Leutenegger CM, Bahula C, Gold P, Hofmann-Lehmann R, Salmons B, Lutz H, Gunzburg WH. Influence of preassay and sequence variations on viral load determination by a multiplex real-time reverse transcriptase-polymerase chain reaction for feline immunodeficiency virus. *J Acquir Immune Defic Syndr* (2001) **26**:8–20. doi:10.1097/00126334-200101010-00002

12. Kubacki J, Fraefel C, Bachofen C. Implementation of next-generation sequencing for virus identification in veterinary diagnostic laboratories. *J Vet Diagn Invest* (2021) **33**:235–247. doi:10.1177/1040638720982630
