# Supplementary material for: Management of Suspected Cases of Feline Immunodeficiency Virus Infection in Eurasian Lynx (Lynx lynx) During an International Translocation Program
Source: Front Vet Sci. 2021 Oct 25;8:730874. doi: 10.3389/fvets.2021.730874 (PMC8573149; doi:10.3389/fvets.2021.730874)
Supplement: Supplementary file 3 [file Data_Sheet_3.docx]

Supplementary Material

# 3 Blood values

**Table 2** Blood chemistry and hematological values outside reference intervals (unpublished author data), as indicated by the arrows (↑ increased; ↓ decreased) of the three FIV-positive Eurasian lynx (*Lynx lynx*) at different time points: 1^st^ capture (C1), 2^nd^ capture (C2), during quarantine (Q) and before euthanasia (E). Empty cells: no abnormality (values within normal range).

| Abnormality^a^ | ADIN | | | NAIA | | | | SENI | | | |
| --- | --- | --- | --- | --- | --- | --- | --- | --- | --- | --- | --- |
|  | **C1** | **C2** | **E** | **C1** | **C2** | **Q** | **E** | **C1 (n/a)** | **C2** | **Q** | **E** |
| Hematocrit (%) |  |  | 32↓ |  |  |  |  |  |  | 37↓ | 37↓ |
| Hemoglobin (g/dl) |  |  | 10↓ |  |  |  |  |  |  |  |  |
| MCH (pg) |  | 17.9↑ |  |  |  |  |  |  |  |  |  |
| MCHC (g/dl) |  |  |  |  |  | 35↑ | 37↑ |  |  | 36↑ | 36↑ |
| Erythrocytes (*10E6/ul) |  | 7.44↓ | 6.62↓ |  |  |  |  |  |  |  |  |
| Leucocytes (310E3/ul) |  |  |  |  |  |  |  |  |  | 37.9↑ | 21.1↑ |
| Neutrophiles (310E3/ul) |  |  |  |  |  |  |  |  |  | 34.09↑ | 18.74↑ |
| Monocytes (310E3/ul) |  |  |  |  |  |  |  |  |  | 1.46↑ |  |
| Total proteins (g/l) |  |  |  | 86.3↑ |  |  |  |  |  |  |  |
| Creatinine (umol/l) |  |  |  |  |  | 180↑ | 182↑ |  |  |  |  |
| Cholesterol (mmol/l) |  |  | 4.2↑ |  |  | 1.7↓ | 1.3↓ |  |  |  | 1.9↓ |
| ASAT (U/l) |  |  |  |  |  |  |  |  |  | 516↑ |  |
| ALAT (U/l) |  |  |  |  |  |  |  |  |  | 357↑ |  |
| Calcium (mmol/l) |  | 2.28 ↓ |  |  |  |  |  |  | 2.23 ↓ |  |  |

n/a, not available; MCH, mean corpuscular hemoglobin; MCHC, mean corpuscular hemoglobin concentration; ASAT, Aspartate-Aminotransferase; ALAT, Alanine-Aminotransferase

^a^Values within the normal range in all three lynx included: mean corpuscular volume (MCV), red cell distribution width (RDW), basophiles, lymphocytes, urea, bilirubin, triglycerides, alkaline phosphatase, lipase, creatine kinase, natrium, potassium, phosphate.
